# ^13^C metabolic flux analysis on roles of malate transporter in lipid accumulation of *Mucor circinelloides*

**DOI:** 10.1186/s12934-019-1207-9

**Published:** 2019-09-10

**Authors:** Lu Wang, Huaiyuan Zhang, Yao Zhang, Yuanda Song

**Affiliations:** 1 Colin Ratledge Center for Microbial Lipids, School of Agricultural Engineering and Food Science, Shandong University of Technology, 266 Xincun West Road, Zibo, 255000 Shandong People’s Republic of China; 2 Key Laboratory of Shandong Provincial Universities for Technologies in Functional Agricultural Products, Shandong University of Technology, 266 Xincun West Road, Zibo, 255000 People’s Republic of China

**Keywords:** ^13^C metabolic flux analysis, Malate transporter, Lipid accumulation, *Mucor circinelloid*es

## Abstract

**Background:**

Mitochondrial and cytoplasmic malate transporter proteins are responsible for transmembrane transport of malate, thereby linking malate metabolism in various subcellular regions of the cell. These transporters play an important role in fatty acid biosynthesis of oleaginous microorganisms. Our previous studies have found that lipid content of the recombinant *Mucor circinelloid*es (*M. circinelloid*es) strain with mitochondrial malate transporter (*mt*) gene overexpression was increased by 70%, while that of strain with *mt* gene knockout was decreased by 27%. However, the mechanism of malate transporter promoting the transport of mitochondrial malate and citrate related to lipid accumulation is not clear. Therefore, ^13^C-labeled glucose metabolic flux analysis was carried out to identify the metabolic network topology and estimate intracellular fluxes of genetically engineered *M. circinelloides* strains for the purpose of better understanding the roles of malate transporters in citrate transport systems and lipid accumulation.

**Results:**

The metabolic flux distribution analysis suggested that tricarboxylic acid (TCA) cycle flux ratio of *mt*-overexpression strains was decreased compared to that of the control strain, but in contrast, glyoxylic acid (GOX) cycle flux ratio was increased. Accordingly, the *mt*-knockout strain showed an opposite phenomenon with a higher TCA cycle flux ratio and a lower GOX cycle flux ratio than the control strain. GOX cycle might be more effective than TCA cycle in producing malate and oxaloacetate replenishment. Moreover, a relatively higher flux ratio of the pentose phosphate (PP) pathway was obtained in *mt*-overexpression strains, but no significant difference in the malic enzyme flux between recombinant strains and the control strain. Our results confirmed that PP pathway might play an important role for supplying NADPH and malic enzyme is not a limiting factor for fatty acid synthesis in oleaginous fungus *M. circinelloides* strains.

**Conclusion:**

Intracellular metabolic flux information suggested that *mt*-overexpression strains had higher flux in PP pathway and GOX cycle, lower flux in TCA cycle, and no difference in malic enzyme cycle. Together, the role of malate transporter was assumed to further participate in transporting cycle of acetyl-CoA and drive PP pathway to supply NADPH required for lipid accumulation in recombinant *M. circinelloides* strains.

## Background

Oleaginous microorganism can synthesize a large amount of triglycerides, accounting for 20–80% of cell dry weight (w/w) [1]. Among them, filamentous fungi and microalgae can synthesize functional long-chain polyunsaturated fatty acids (PUFAs), which are beneficial to human health and have been identified as important nutrient food resources [2, 3], such as γ-linolenic acid (GLA), eicosapentaenoic acid (EPA), docosahexaenoic acid (DHA), etc. Other oleaginous microorganism, like yeast, microalgae and some bacteria, can synthesize common lipids which are potentially an important and attractive substitute for fossil fuels [4–7].

So far, a lot of research on microbial lipid accumulation have been done, and a clear understanding of the biochemical mechanism of lipid accumulation after nitrogen depletion has been elucidated [8]. For eukaryotes, the final product of glycolysis, pyruvate, enters the mitochondria and then is converted to an important intermediate metabolite, acetyl-CoA, which must be transported from the mitochondrion to the cytosol for lipid synthesis. Therefore, the mitochondrial citrate transport system that transports acetyl-CoA (citrate as its acetyl carrier) is not only the link between glucose metabolism and lipid synthesis, but also an important factor affecting the lipid synthesis in eukaryotes. This citrate transporter which is located on the mitochondrial inner membrane, acts as the carrier of citrate and malate to shuttle between cytosol and mitochondria [9]. Previously, the relationship between the activity of citrate transporters and lipid synthesis has been studied in oleaginous yeast [10] or non-oleaginous yeast [11] and mammal [12, 13]. Mitochondrial citric acid efflux rate of oleaginous yeast was 2.5-fold higher than that of non-oleaginous yeast, while the *K*_*m*_ value of transporters in non-oleaginous yeast was 2-fold higher than that of oleaginous yeast [10, 11, 14]. Exhaustive studies on mammalian citrate transporters have demonstrated that the activity of citrate transporter is decreased in cases of diabetes and hungry, whereas this activity is raised in cases of insulin addition and feeding [15]. All of above results suggested that the regulation of the citrate transporter activity is closely related to lipid synthesis. However, the function and mechanism of mitochondrial citrate transport system in oleaginous filamentous fungi is still far from clear.

Another important transporter which has a significant effect on citrate transfer in mitochondria is malate transporter. The correlation between the malate transporter and lipid accumulation in *M. circinelloides* was preliminarily studied by knockout and overexpression of the malate transporter (*mt*) gene [16]. Our results showed that the fatty acid content of the recombinant strain with overexpression of *mt* was increased by 70%, while that of the strain with knockout *mt* was decreased by 27% [17]. Although the role of malate transporter in promoting fatty acid synthesis has been revealed, the mechanism behind malate transporter promoting the transport of mitochondrial malate and citrate related to lipid accumulation is still not clear. In order to better understand the regulation mechanism of the malate transporter involved in lipid accumulation, in the present study, the intracellular metabolic fluxes of genetically engineered *M. circinelloides* strains were investigated using ^13^C-labeled metabolic flux analysis. This study showed for the first time, the quantitative analysis of the changes in intracellular metabolic flux of these engineered strains to estimate and understand the role and mechanism of the malate transporter involved in lipid metabolism.

## Results and discussion

### Fermentation profiles and the establishment of a metabolic steady state

**Fig. 1:**
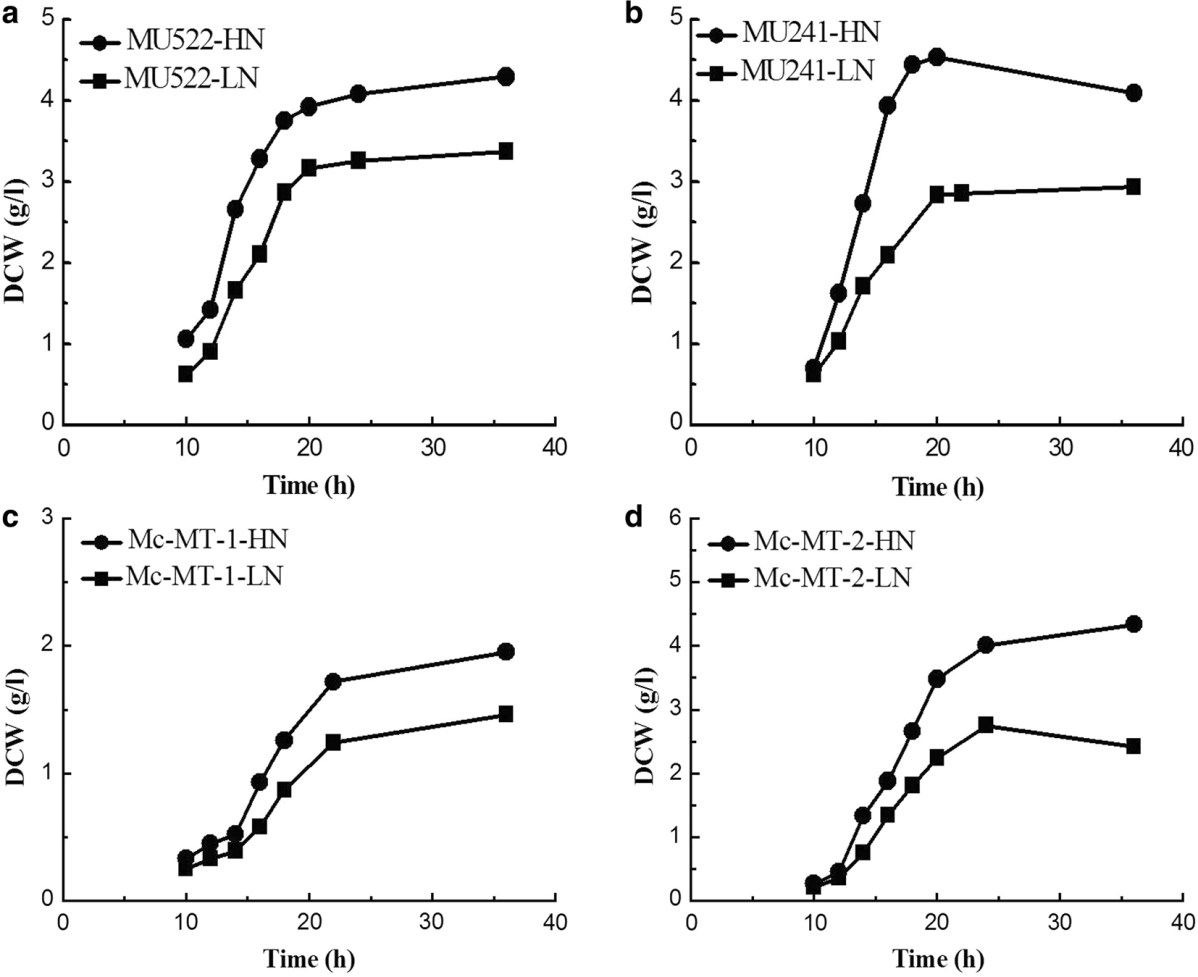
Growth curve of recombinant *M. circinelloides* strains under different nitrogen concentrations. **a** *mt*-knockout strain MU522. **b** Wide type strain MU241, as the control. **c** *mt*-overexpression strain Mc-MT-1. **d** *mt*-overexpression strain Mc-MT-2

**Fig. 2:**
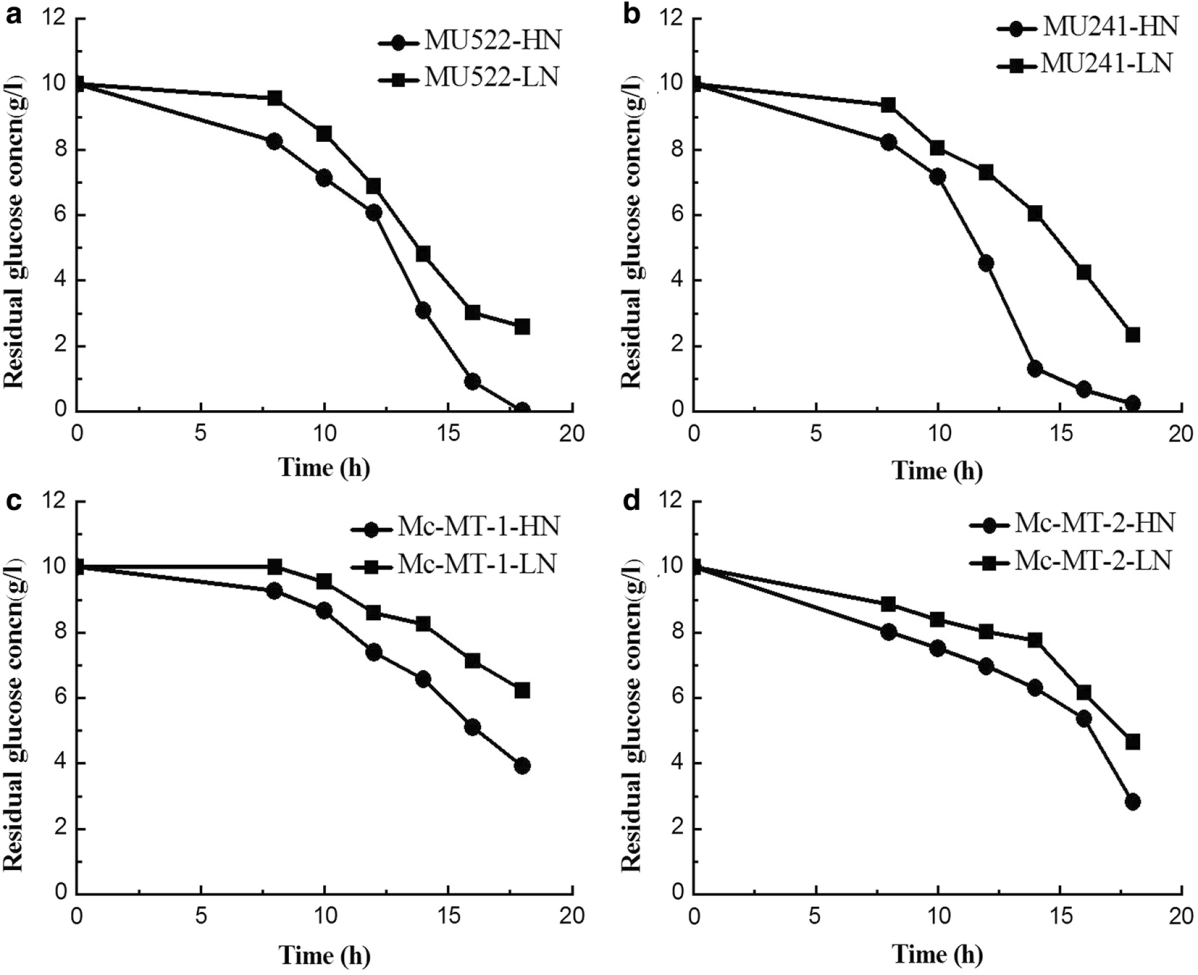
Residual glucose concentration of recombinant *M. circinelloides* strains in different nitrogen culture supernatant. **a** *mt*-knockout strain MU522. **b** Wide type strain MU241, as the control. **c** *mt*-overexpression strain Mc-MT-1. **d** *mt*-overexpression strain Mc-MT-2

**Fig. 3:**
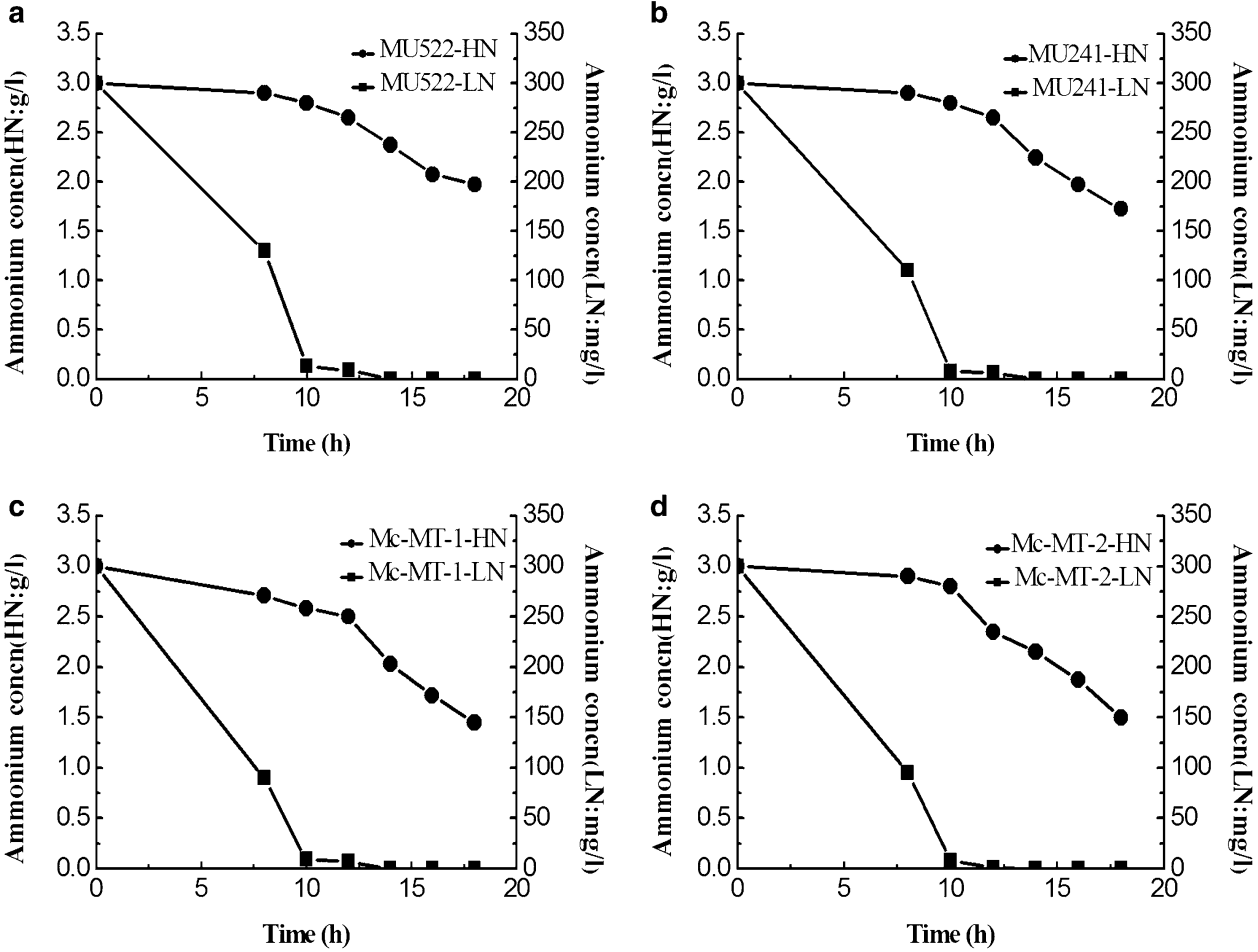
Residual Ammonium ion concentration of recombinant *M. circinelloides* strains in different nitrogen culture supernatant. **a** *mt*-knockout strain MU522. **b** Wide type strain MU241, as the control. **c** *mt*-overexpression strain Mc-MT-1. **d** *mt*-overexpression strain Mc-MT-2

**Fig. 4:**
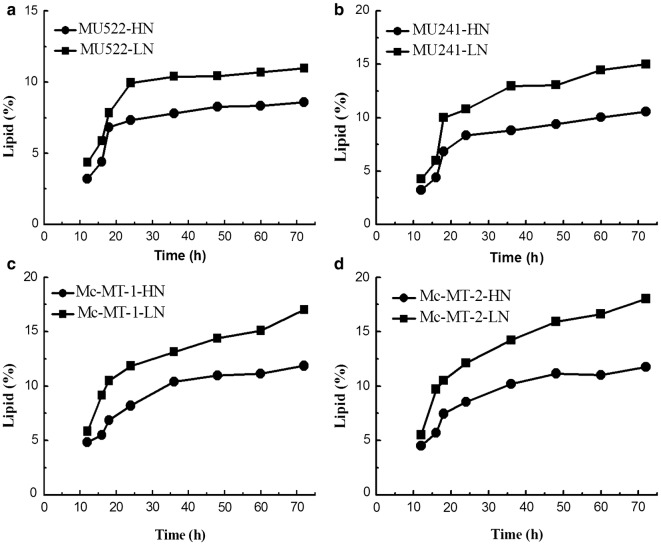
Lipid accumulation curve of recombinant *M. circinelloides* strains under different nitrogen concentrations. **a** *mt*-knockout strain MU522. **b** Wide type strain MU241, as the control. **c** *mt*-overexpression strain Mc-MT-1. **d** *mt*-overexpression strain Mc-MT-2

Previously, the malate transporter (*mt*) gene was overexpressed or knocked out in *M. circinelloides* to construct recombinant strains, which are as follows: MU522 (*mt*-knockout, CCTCC M 2015552), MU241 (wild-type, as the control) and Mc-MT (*mt*-overexpression, CCTCC M 2015551). The transformants of *mt*-overexpressing gene were named Mc-MT-1 and Mc-MT-2, respectively [16]. These engineered strains were respectively inoculated in high nitrogen (HN) and low nitrogen (LN) modified K & R medium with glucose as the sole carbon source. Typical culture profiles of the strains under two different nitrogen conditions were shown in Fig. 1. Generally, all of the strains exhibited similar growth profiles for a 10 h lag phase and an balance phase from 12 h to 20 h in both culture conditions. However, due to nitrogen limitation, the final cell concentrations (dry cell weight, DCW) of each strain in LN medium was obviously lower than that grown in HN medium. Not surprisingly, specific growth rates of these recombinant strains under HN condition improved by 45.5–126.9% compared to that under LN condition at the balanced growth phase (Table 1). It is worth to note that, the DCW and growth rate of one of *mt*-overexpressing strains (Mc-MT-1) were far less than other strains under both nitrogen concentrations (Fig. 1 and Table 1), probably due to the low cell viability of this transformant. However, considering that this strain has a relatively high lipid content (Fig. 4) and lipid production rate (Table 1), we still carry out follow-up studies on this strain. Glucose consumption profiles also differed depending on nitrogen concentration, and consequently, nitrogen limitation resulted in a decreased glucose uptake rate by 30.9%-92.2% when compared to that with sufficient nitrogen (Fig. 2). The NH_4_^+^ concentrations in the cultures of the recombinant strains were also detected and the results showed that NH_4_^+^ slumped markedly and exhausted within 10 h in LN condition (Fig. 3). It is generally believed that oil-producing fungi can accumulate large amounts of lipids when carbon sources are sufficient and other nutrient elements (such as nitrogen sources) are lacking [17]. Therefore, lipid accumulation profiles of the recombinant strains were investigated during culture process, respectively (Fig. 4). As shown in Fig. 4, the lipid contents of all the recombinant strains in LN condition were significantly higher than that in HN condition, which was identical with the lipid production rates (Table 1). It should be noted that during the whole fermentation process, very a little difference in lipid content among recombinant strains can be seen at the balanced growth phase since the carbon source is mainly used for growth; while at the late stationary phase, the cells no longer grow, and the carbon source is mainly used for accumulating lipids and then apparent difference in lipid content could be displayed. The abovementioned physiological results in *M. circinelloides* strains under different nitrogen conditions were consistent with our previous report [18].

**Table 1:** Specific rates of recombinant *M. circinelloides* strains under different nitrogen concentrations at a metabolic steady state

Strains	Condition	Cell growth rate (h^−1^)	Glucose consumption rate (mmol/g/h)	Lipid production rate (mmol/g/h)
MU522	HN	0.46 ± 0.02	7.16 ± 0.31	0.96 ± 0.05
	LN	0.30 ± 0.01	5.36 ± 0.19	1.00 ± 0.03
MU241	HN	0.59 ± 0.03	8.15 ± 0.35	1.28 ± 0.06
	LN	0.26 ± 0.01	4.24 ± 0.11	2.06 ± 0.07
Mc-MT-1	HN	0.16 ± 0.01	3.69 ± 0.17	1.33 ± 0.04
	LN	0.11 ± 0.01	2.82 ± 0.09	2.13 ± 0.08
Mc-MT-2	HN	0.40 ± 0.02	6.56 ± 0.30	1.28 ± 0.05
	LN	0.26 ± 0.01	4.31 ± 0.15	2.26 ± 0.08

Each experiment was performed in triplicate, and all data are reported as means ± standard deviations

*HN*, high nitrogen, *LN*, low nitrogen

In batch cultures, a metabolic steady state exists during balanced growth where the growth rate is constant. Currently, isotope steady state is a prerequisity for all the mathematical frameworks that are publicly available for ^13^C-labeled flux analysis [19]. These preconditions indicate that all intermediate concentrations and fluxes remain constant throughout the ^13^C-labeled flux experiment. The growth tendency of recombinant strains were similar in HN or LN mediums, but the time points in balanced growth of these strains were not completely consistent, which were 14–18 h. Therefore, the metabolic steady state of 14–18 h was chosen as the time point for the determination of biomass macromolecular compositions and ^13^C metabolic flux analysis.

### Biomass composition of recombinant *M. circinelloides* strains

In order to calculate intracellular absolute fluxes, the amount of metabolites extracted to meet biosynthesis demands should be known. The contents of cellular units (carbohydrate, lipid, protein, DNA and RNA) which accounts for the majority of the total dry cell weight, were analyzed and were listed in Table 2. Early research showed that lipid begin to accumulate after nitrogen depletion [20], and so the total lipid content of all strains under LN condition were higher than that of the HN condition. With the prominent weaken in nitrogen concentration, the lipid content increased in both *mt*-overexpression strains, but it remained almost unvaried in *mt*-knockout strain under two different nitrogen concentrations. In balanced phase under LN condition, the lipid content of *mt*-overexpression strains had an increase of 22% and 27% compared with the control strain, whereas the *mt*-knockout strain had a decreased lipid content of 23%. And also, carbohydrate and protein contents differed generally between LN and HN condition as well. The carbohydrate and protein contents were similar between recombinant strains under identical nitrogen concentration. Nitrogen limitation has no remarkable effect on DNA or RNA levels, and no available differences were obtained from our results.

**Table 2:** Macromolecular compositions in recombinant *M. circinelloides* strains under different nitrogen concentrations

Strains	Condition	Biomass composition %					
		Protein	Carbohydrate	Lipid	DNA	RNA	Others
MU522	HN	59.6 ± 2.5	10.6 ± 0.4	6.6 ± 0.2	3.7 ± 0.2	7.5 ± 0.3	12.0 ± 0.1
	LN	54.6 ± 0.9	24.7 ± 1.3	7.8 ± 0.3	1.6 ± 0.1	4.5 ± 0.0	6.8 ± 0.2
MU241	HN	56.5 ± 1.8	7.0 ± 0.3	7.4 ± 0.2	2.4 ± 0.1	8.2 ± 0.1	18.5 ± 0.7
	LN	45.1 ± 2.1	27.6 ± 0.8	10.1 ± 0.1	2.0 ± 0.1	3.9 ± 0.1	11.3 ± 0.2
Mc-MT-1	HN	56.2 ± 2.4	9.5 ± 0.5	7.3 ± 0.3	6.7 ± 0.3	7.6 ± 0.3	12.7 ± 0.5
	LN	42.5 ± 1.9	26.4 ± 1.1	12.3 ± 0.5	4.5 ± 0.1	5.2 ± 0.2	9.1 ± 0.2
Mc-MT-2	HN	53.8 ± 1.7	12.0 ± 0.6	7.6 ± 0.4	2.7 ± 0.1	5.6 ± 0.1	17.3 ± 0.3
	LN	44.8 ± 1.1	26.6 ± 1.5	12.8 ± 0.3	3.1 ± 0.1	5.4 ± 0.1	7.3 ± 0.1

*HN* high nitrogen, *LN* low nitrogen

The main fatty acid composition of recombinant *M. circinelloides* strains were palmitic acid (C16:0) and octadecenoic acid (C18:1, C18:2 and C18:3), which were basically consistent with previous study [21]. Overall, there was no significant diversity in the composition of fatty acids among recombinant strains between high nitrogen and low nitrogen conditions (shown in Table 3). Nitrogen limitation increased the content of saturated fatty acid (palmitic and stearic acids) but decreased the content of linoleic acid.

**Table 3:** Fatty acid profile in recombinant *M. circinelloides* strains under different nitrogen concentrations

Strains	Condition	Fatty acid profile						
		14:0	16:0	16:1	18:0	18:1	18:2	18:3
MU522	HN	2.75 ± 0.01	20.31 ± 0.56	3.71 ± 0.21	3.70 ± 0.05	18.53 ± 0.55	21.05 ± 0.39	29.96 ± 0.88
	LN	3.01 ± 0.05	24.45 ± 0.27	3.79 ± 0.39	9.26 ± 0.15	17.37 ± 0.46	18.83 ± 1.03	23.29 ± 0.55
MU241	HN	1.98 ± 0.06	22.32 ± 0.68	2.16 ± 0.11	3.58 ± 0.27	18.27 ± 0.75	21.39 ± 0.45	30.30 ± 1.44
	LN	2.51 ± 0.03	24.05 ± 0.56	2.21 ± 0.23	8.97 ± 0.19	17.87 ± 0.70	19.40 ± 0.37	24.99 ± 0.62
Mc-MT-1	HN	2.73 ± 0.06	25.36 ± 0.63	1.86 ± 0.11	3.90 ± 0.26	13.25 ± 0.36	18.02 ± 1.20	34.89 ± 0.85
	LN	2.61 ± 0.39	27.53 ± 0.80	1.58 ± 0.89	7.38 ± 0.11	14.19 ± 0.21	16.19 ± 0.55	30.53 ± 0.71
Mc-MT-2	HN	2.01 ± 0.02	22.80 ± 0.17	1.72 ± 0.14	2.48 ± 0.04	18.91 ± 0.74	21.73 ± 0.47	30.35 ± 1.20
	LN	1.91 ± 0.11	27.53 ± 1.14	1.11 ± 0.01	6.36 ± 0.04	17.35 ± 1.25	20.00 ± 0.58	25.74 ± 0.82

*HN*, high nitrogen, *LN* low nitrogen

Amino acids compositions in recombinant strains under high nitrogen and low nitrogen conditions are shown in Table 4, and a total of 17 amino acids were detected. This is because asparagine and glutamine were easily oxidized in the hydrolysis process and then converted into aspartic acid and glutamic acid, respectively, and the final calculation was based on the sum of the two amino acids. It has been reported that tryptophan is easily oxidized during proteolysis [22], which was also indeed not detected in this experiment. The contents of all amino acids were almost similar in the medium with nitrogen-sufficient or nitrogen-limited conditions. The amino acid composition proportion of recombinant strains was not significantly different compared to the control strain (Table 4).

**Table 4:** Amino acids composition in recombinant *M. circinelloides* strains under different nitrogen concentrations

Amino acid	MU522		MU241		Mc-MT-1		Mc-MT-2	
	HN	LN	HN	LN	HN	LN	HN	LN
Asp	0.1100	0.1242	0.1098	0.1134	0.1463	0.1341	0.1190	0.1259
Glu	0.1515	0.1510	0.1458	0.1456	0.1372	0.1409	0.1581	0.1497
Ser	0.0350	0.0384	0.0359	0.0366	0.0340	0.0337	0.0348	0.0350
His	0.0266	0.0287	0.0256	0.0262	0.0267	0.0287	0.0251	0.0254
Gly	0.0566	0.0582	0.0581	0.0570	0.0516	0.0526	0.0547	0.0549
Thr	0.0474	0.0491	0.0472	0.0425	0.0443	0.0455	0.0464	0.0476
Arg	0.0621	0.0578	0.0634	0.0552	0.0761	0.0631	0.0669	0.0668
Ala	0.0991	0.0754	0.0894	0.0766	0.0715	0.0883	0.0832	0.0785
Tyr	0.0270	0.0270	0.0259	0.0333	0.0241	0.0245	0.0270	0.0285
Cys-s	0.0016	0.0022	0.0015	0.0017	0.0015	0.0021	0.0019	0.0031
Val	0.0658	0.0653	0.0666	0.0758	0.0612	0.0615	0.0636	0.0639
Met	0.0250	0.0211	0.0236	0.0224	0.0217	0.0209	0.0235	0.0232
Phe	0.0475	0.0511	0.0483	0.0477	0.0451	0.0444	0.0453	0.0452
Ile	0.0540	0.0563	0.0548	0.0553	0.0514	0.0518	0.0537	0.0535
Leu	0.0747	0.0783	0.0771	0.0774	0.0725	0.0723	0.0729	0.0728
Lys	0.0827	0.0807	0.0869	0.0770	0.0962	0.0980	0.0861	0.0920
Pro	0.0334	0.0354	0.0402	0.0565	0.0386	0.0375	0.0378	0.0340

*HN* high nitrogen, *LN* low nitrogen

### Metabolic flux distribution associated with lipid accumulation in recombinant *M. circinelloides* strains

^13^C metabolic flux analysis is currently the preferred method in microbial [23], mammalian [24], and plant [25] systems. In the present study, ^13^C metabolic flux analysis was implemented on recombinant *M. circinelloides* strains to analyze the effects of overexpression or knockout of malate transporter gene on metabolic fluxes using the calculated extracellular flux values and GC–MS metabolite labeled data as inputs. A large amount of data on isotopically labeled cell metabolite isomer distribution obtained by ^13^C labeled experiments in conjunction with mass spectrometry was shown in Additional file 1: Table S1. GC–MS analysis of the glucose tracer demonstrated that each tracer had an isotope purity of about 20%, which was in accordance with the culture conditions [26]. Bioreaction network of the central carbon metabolism of *M. circinelloides* is required for the flux analysis. All the biochemical reactions and its atom transitions involved in the network are listed in Additional file 2: Table S2. ^13^C metabolic flux analysis calculations based on the elementary metabolite units were performed using Metran software [22]. For all cultures, the glucose consumption was defined as a value of 100 to facilitate the comparison of intracellular metabolic flux distributions between recombinant strains and the control strain.

**Fig. 5:**
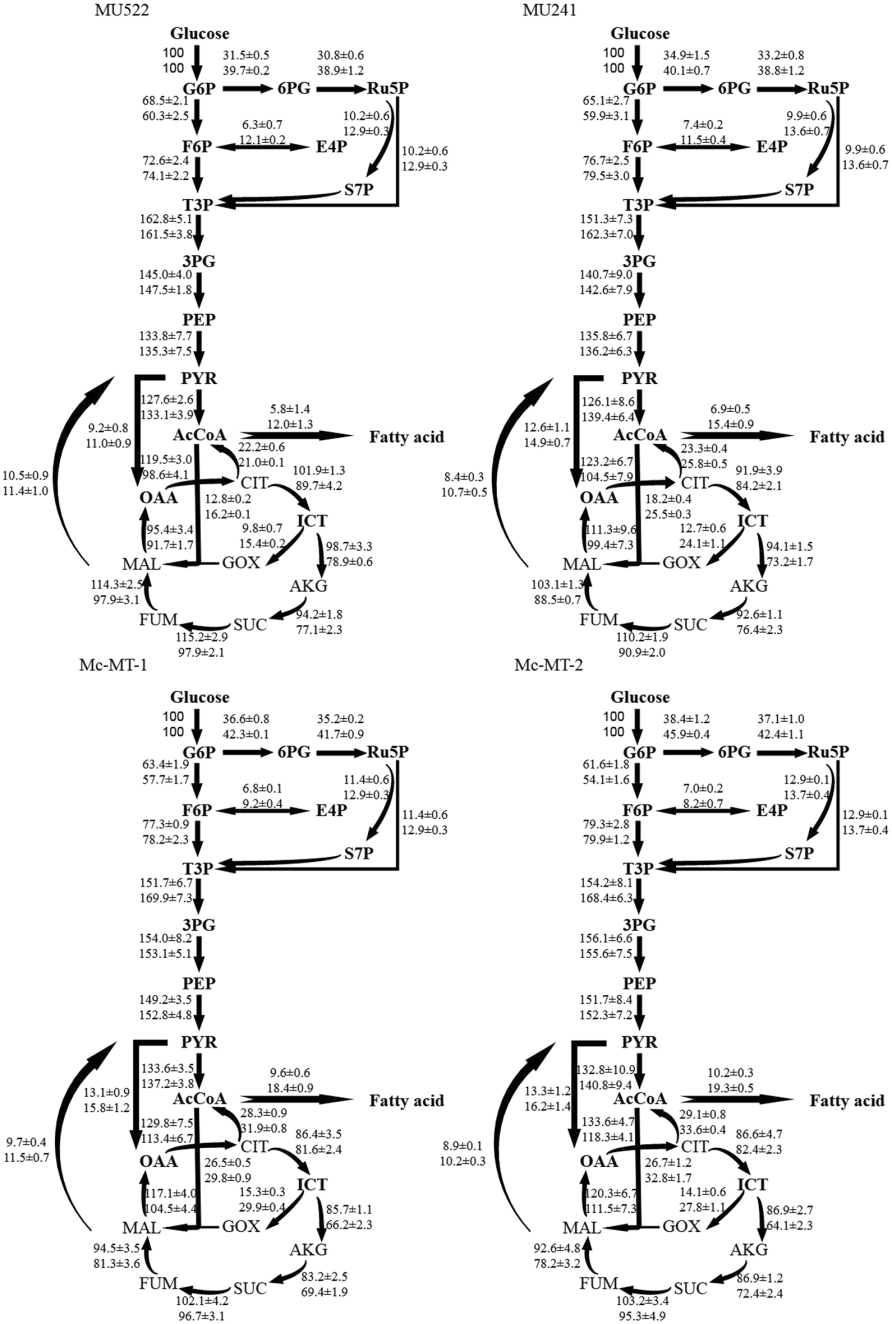
Metabolic fluxes in recombinant *M. circinelloides* strains under different nitrogen concentrations. The top fluxes represent under high nitrogen (HN) culture condition, and the bottom fluxes represent under low nitrogen (LN) culture condition. *G6P*, glucose-6-phosphate, *6PG*, 6-phosphogluconate, *Ru5P*, ribulose 5-phosphate, *F6P*, fructose-6-phosphate, *E4P*, erythrose-4-phosphate, *S7P*, sedoheptulose-7-phosphate, *T3P*, triose-3-phosphate, *3PG*, 3-phosphoglycerate, *PEP*, phosphoenolpyruvate, *PYR*, pyruvic acid, *OAA*, oxaloacetic acid, *CIT*, citrate, *ICT*, isocitric acid, *AKG*, α-ketoglutaric acid, *SUC*, succinic acid, *FUM*, fumaric acid, *MAL*, malate, *GOX*, glyoxylate

The estimated metabolic fluxes of recombinant strains cultured in HN and LN conditions were shown in Fig. 5. It clearly displayed that the intracellular fluxes changed during the balanced growth phase. Because lipids are highly reduced substance, triacylglycerol overproduction requires large quantities of NADPH. A recent analysis showed that the oxidative PP pathway is the primary source of lipogenic NADPH in *Yarrowia lipolytica* [27]. And our previous results confirmed that the reactions of PP pathway would appear to be the most likely route providing NADPH in oleaginous fungus *M. circinelloides* [18]. Ratledge also used stoichiometric analysis to confirm that the PP pathway might be an important source of NADPH in lipid synthesis [28]. As shown in Fig. 5, the flow ratios into the PP pathway of all the recombinant strains cultured in LN condition, were higher than that cultured in HN condition with the increase varies from 14.9% to 26.0%, which might be associated with higher lipid accumulation in this fungi. In the process of lipid accumulation (nitrogen limitation), the carbon flux of the control strain through the EMP and PP pathway accounted for 59.9% and 40.1% of the glucose uptake. Our results showed that there was no significant difference in PP pathway between *mt*-knockout strain and the control strain under LN condition. However, compared with the control strain, the flux ratios of EMP/PP pathway in *mt*-overexpression strains were decreased to 57.7/42.3 (for Mc-MT-1) and 54.1/45.9 (Mc-MT-2), respectively, when cultured under nitrogen-limited medium. Simultaneously, when compared with control stain, 5.5% (for Mc-MT-1) and 14.5% (Mc-MT-2) higher flux of PP pathway in *mt*-overexpression strains were obtained in LN condition. Therefore, our results indicated that a relatively higher flux ratio of PP pathway, or a lower EMP/PPP ratio is beneficial to lipid accumulation in these recombinant *M. circinelloides* strains. NADPH generation in the entire central carbon metabolism indeed affects lipid production since lipid biosynthesis requires NADPH as a cofactor [28]. NADPH production pathways in cells, such as the PP pathway, might be an important provider of NADPH required for lipid synthesis [28]. Several oil-producing microalgae [29] and lipogenic yeast [27] and our previous [18] and present metabolic flux analysis of *M. circinelloides* intuitively showed that the PP pathway might provide higher proportions of NADPH for lipid biosynthesis.

We also found that the main energy producing pathway, TCA cycle in all of the recombinant strains under LN condition were decreased than that in HN condition, which were consistent with our previous studies [18, 30]. The biochemical mechanism from nitrogen restriction to the retardation of TCA cycle has been clearly elucidated by previous researchers [31]. In addition, the TCA cycle flux ratios in *mt*-overexpression strains were significantly lower than the control strain, and conversely the *mt*-knockout strain appeared an obviously higher TCA cycle flux than the control stain whether grown under HN or LN condition. Our results suggested that the overexpression of malate transporter gene might accelerate the transport system of TCA to obtain more intermediate substrates which eventually led to an association with high lipid accumulation in *M. circinelloides*.

An interesting phenomenon was obtained that the flux ratios of GOX cycle (the bypass in the TCA cycle from isocitrate to malate to participate in the citrate cycle) in *mt*-overexpression strains were significantly higher than the control strain, and conversely, the GOX flux in *mt*-knockout strain was apparently lower than the control strain regardless of the HN or LN media. Especially under LN condition, the GOX fluxes were improved by 24.1% (in Mc-MT-1) and 15.4% (in Mc-MT-2), respectively when compared with the control strain, while in *mt*-knockout strain (MU522) the GOX flux decreased by 36.1% than the control stain. This finding is consistent with early biochemical studies in *M. circinelloides* WJ11 which also has a low flux of TCA cycle but a high flux of GOX cycle associated with high lipid accumulation [18]. It is assumed that during the process of lipid biosynthesis, some of accumulated citrate is transported out of the mitochondria by the carrier to be lysed into acetyl-CoA and oxaloacetate [1], while other citrate is converted into isocitrate, which could be metabolized through TCA cycle or the GOX cycle. Malate obtained from oxaloacetate driven by malate dehydrogenase could then be decarboxylated to generate pyruvate via the NADP^+^ dependent cytosolic malic enzyme and in this process an adequate supply of NADPH might be also produced for lipid synthesis [28]. But *M. circinelloides* original strains used in this study were known to have relatively low NADP^+^ dependent malic enzyme activity [18] which we have also verified. In other words, even if the malate transporter transported a large amount of malate, due to the low activity of malic enzyme, it is impossible to catalyze the conversion of malate to pyruvate for fatty acid synthesis. And so there is no significant difference in the malic enzyme flux between *mt*-knockout strain or *mt*-overexpression strain and the control strain. However, the availability of cytosolic NADH is limited, and recombinant *M. circinelloides* strains have relatively low NADP^+^ dependent malic enzyme activity, and thus a synergic pathway such as the PP pathway in producing NADPH would be necessary. This also indicates that the PP pathway might provide the majority of NADPH for lipid biosynthesis during low lipid production stage in recombinant *M. circinelloides* strains. Under the action of malate transporter, malate is transported back into the mitochondria, and afterwards regenerated into oxaloacetate by the catalysis of malate dehydrogenase. The internal transportation of malate functioned by malate transporter to generate oxaloacetate, which allows for further involvement in transporting cycle of acetyl-CoA. And this makes it possible to provide more sufficient acetyl-CoA for fatty acid synthesis. Our results have determined that GOX pathway is more effective than TCA cycle in producing malate and oxaloacetate replenishment. Previously, it has been reported that GOX cycle could reduce glucose uptake by increasing the cytosolic malate flux to generate additional malate [32], which is in accordance with our results. Indeed, mitochondrial malate/citrate transporter proteins might be responsible for the internal and external transport of malate/citrate [33], and extensive study has to be implemented to explore the role of GOX associated with malate/citrate transporter in lipid accumulation of oleaginous fungus.

### Yield and consumption of NADPH in recombinant *M. circinelloides* strains

As a prerequisite for fatty acid synthesis, the supply of NADPH and acetyl-CoA plays an important role in the lipid accumulation process of oleaginous microorganisms. Thus, NADPH is particularly essential as the source of reducing power required for fatty acid synthesis in oleaginous fungus. The main pathways for NADPH generation during glucose metabolism in oleaginous fungi include: the PP pathway with glucose 6-phosphate dehydrogenase (G6PD) and 6-phosphogluconate dehydrogenase (6PGD), or the pyruvate/oxaloacetate/malate (POM) cycle through NADP^+^ dependent malic enzyme, or the TCA cycle via NADP^+^ dependent isocitrate dehydrogenase (ICDH). In the present study, the yield and consumption of NADPH in recombinant *M. circinelloides* strains under HN and LN conditions were, respectively shown in Table 5. It was found that slightly higher proportions of NADPH required for lipid synthesis in all of the recombinant strains under HN or LN condition were generated from PP pathway by G6PD and 6PGD, but not malic enzyme and ICDH when compared with that of the control strain (Table 5). When *mt*-overexpression strains were cultured under LN medium, the NADPH generated from G6PD were improved by 5.5% for Mc-MT-1 and 14.5% for Mc-MT-2 than the control strain, respectively. These results suggested that PP pathway might play a vital role for the provision of NADPH for lipid accumulation in *M. circinelloides*, which was a little different from previous studies [18]. As known, malic enzyme was originally thought to be a major provider of NADPH during fatty acid synthesis and regulates the synthesis of fatty acids [34]. However, the latest researches raise questions about the role of malic enzyme in the process of lipid accumulation, and Ratledge [28] had also theoretically analyzed the contribution of NADPH generating pathways and through chemical calculations found that NADPH provided by malic enzyme is not enough to complete lipid accumulation; the reaction of PP pathway would appear to be most like route and there is a possibility of some NADPH generated from cytosolic ICDH reaction. But our results confirmed that PP pathway might play an important role for supplying NADPH and malic enzyme is not a limiting factor for fatty acid synthesis in oleaginous fungus *M. circinelloides* strains.

**Table 5:** Yield and consumption of NADPH in recombinant *M. circinelloides* strains under different nitrogen concentrations

Strains	Condition	NADPH yield				NADPH consumption		
		PP pathway		POM cycle	TCA cycle	Biosynthesis		
		G6PD	6PGD	ME	ICDH	Lipid	Protein	Nucleic acid
MU522	HN	0.315	0.308	0.105	0.987	0.127	1.514	0.075
	LN	0.397	0.389	0.114	0.789	0.146	1.486	0.056
MU241	HN	0.349	0.332	0.084	0.941	0.141	1.482	0.082
	LN	0.401	0.388	0.107	0.732	0.213	1.361	0.056
Mc-MT-1	HN	0.366	0.352	0.097	0.857	0.135	1.473	0.066
	LN	0.423	0.417	0.115	0.662	0.238	1.321	0.059
Mc-MT-2	HN	0.384	0.371	0.089	0.869	0.144	1.489	0.084
	LN	0.459	0.424	0.102	0.641	0.242	1.331	0.052

Data were obtained from the flux distribution presented in Fig. 5. Values are expressed in mol NADPH/mol glucose consumed

*G6PD* glucose 6-phosphate dehydrogenase, *6PGD* 6-phosphogluconate dehydrogenase, *ME* malic enzyme, *ICDH* isocitrate dehydrogenase

## Conclusion

In this study, ^13^C metabolic flux analysis was performed on the recombinant *M. circinelloides* strains of *mt*-overexpressing and *mt*-knockout. Our results suggested that the *mt*-overexpressing strains had a lower TCA cycle flux ratio and a higher GOX flux ratio when compared with the control strain, and the *mt*-knockout strain had an opposite phenomenon with a higher TCA cycle flux ratio and a lower GOX cycle flux ratio than that of the control strain. GOX pathway might be more effective than TCA cycle in producing malate and oxaloacetate replenishment. The internal transfer of malate driven by malate transporter might further participate in transporting cycle of acetyl-CoA. Therefore, the role of malate transporter was assumed to provide more adequate precursor (acetyl-CoA) for lipid accumulation. Furthermore, a relatively higher flux ratio of PP pathway was obtained in *mt*-overexpression strains, but no significant difference in the malic enzyme flux between recombinant strains and the control strain. These results confirmed that PP pathway might play a major role in supplement of required NADPH and malic enzyme is not a limiting factor for fatty acid synthesis in oleaginous fungus *M. circinelloides* strains.

## Methods

### Strains and medium

The strains used in this experiment are shown in Table 6. Based on the genomic data search and sequences analysis of *M. circinelloides* CBS277.49, a gene (ID180302) encoding malate transporter was found in NCBI and named *mt*. Both null mutant (Mu522) and overexpressing strains (Mc-MT-1 and Mc-MT-2) of this gene were previously constructed in our laboratory [16].

**Table 6:** Strains used in the experiment

Strains	Relevant description	Reference or source
MU241	Wild-type prototrophic strain as the control	Our lab [13]
MU522	*mt*-knockout, CCTCC M 2015552	
Mc-MT-1	*mt*-overexpression, CCTCC M 2015551	
Mc-MT-2		

The gene (ID180302) encoding malate transporter was found in NCBI and named *mt*. Both null mutant (Mu522) and overexpressing strains (Mc-MT-1 and Mc-MT-2) of this gene were previously constructed in our laboratory

HN medium formula: glucose 10 g/l, (NH-_4_)_2_SO_4_ 3 g/l, KH_2_PO_4_ 7 g/l, Na_2_HPO_4_ 2 g/l, MgSO_4_·7H_2_O 1.5 g/l, CaCl_2_·2H_2_O 0.1 g/l, mother liquor 100 μl/l (FeCl_3_·6H_2_O 0.008 g/l, ZnSO_4_·7H_2_O 0.001 g/l, CuSO_4_·5H_2_O 0.0001 g/l, Co(NH_3_)_2_·6H_2_O 0.0001 g/l, MnSO_4_·5H_2_O 0.0001 g/l). The LN medium is the same as the high nitrogen medium except (NH_-4_)_2_SO_4_ 0.3 g/l [18]. As for the ^13^C-labeled medium: (1) 1-^13^C labeled glucose 10 g/l, (2) U-^13^C labeled glucose 2 g/l, unmarked natural glucose 8 g/l, and the remaining ingredients are the same as the above medium.

### Growth conditions

50 μl spore suspensions of recombinant *M. circinelloides* strains were inoculated into a 100 ml flask containing 10 ml medium. All cultures were shaken at 28 °C and 250 rpm. The control strain and three recombinant strains were collected during the metabolic steady state in ^13^C-labeled medium of HN or LN.

### Determination of extracellular metabolite

The cells were harvested at different fermentation times before filtered, then wash them three times with distilled water. Cells were frozen in ultra-low temperature freezer at − 80 °C, followed by freeze drying. DCW was obtained by differential weighing.

Glucose content in supernatant was measured by glucose oxidase–peroxidase (GOD) using glucose measurement kit (Rongsheng Co. Ltd, China) and the depletion of ammonium was determined by indophenol blue spectrophotometric method [35].

### Biomacromolecule quantification

In order to perform ^13^C metabolic flux analysis, it is essential to determine the content and composition ratio of intracellular biomacromolecules.

Total intracellular carbohydrate was determined by dinitrosalicylic acid (DNS) method. In this procedure, a certain amount of freeze-dried powder by adding HCl (10 ml) at a concentration of 6 M and purified water (15 ml) was boiled for 30 min. After cooled to room temperature, the solution was neutralized with NaOH (6 M) and dilute with water to 100 ml.

Total protein content of cell extraction was measured based on BCA kit (Solarbio Science & Technology Co. Ltd, Beijing). Resuspend the frozen pellets in 8 ml of 6 M HCl. Seal the tubes to prevent the volatilization of HCl. Samples were hydrolyzed for 22 h in an oven at 120 °C after the tubes are filled with nitrogen. After cooled to room temperature, the solution was neutralized with 4.8 ml of NaOH (10 M). Then the solution was carefully transferred to a 25-ml volumetric flask and mixed. Samples were fractionated by the double filter paper retaining the supernatant, and then were carried out for analysis.

Genomic DNA was extracted by DNA quick Plant System kit (TIANGEN BIOTECH Co. Ltd, Beijing) obtaining DNA percentage depending on NanoDrop2000. RNA concentration was determined using method of perchloric acid. Cell culture medium (10 ml) was centrifuged for 15 min at 12000×*g*, 4 °C, washing the sediment twice with 400 μl of pre-cooling HClO_4_ (0.7 mM). The procedure was followed to perform digestion in the thermostatic water bath at 37 °C for 1 h by adding 400 μl KOH (0.3 M). After cooled to room temperature, the solution was neutralized with 133 μl HClO_4_ (3 M) followed by centrifuged for 10 min at 10,000×*g*. Then, 533 μl HClO_4_ (0.5 M) was used to wash the cell sediment with continuous centrifugation. Be carefully transferred supernatant to a 2-ml volumetric flask mixed HClO_4_ (0.5 M) after the collected liquid and by the way of centrifuging to remove impurities. RNA concentration was ascertained by NanoDrop2000.

Freeze-dried powder was disrupted into hydrolysis tube containing 2 ml HCl (4 M) and cooked in a water bath at 80 °C for 3–5 h, and vibrated every 30 min. The pentadecanoic acid (C15:0, Sigma) was added as an internal standard, then lipids were transesterified to fatty acid methyl esters (FAMEs) by addition of 1 ml Hydrochloric acid methanol (10%). The total lipid was measured by the differential weight method. The principal fatty acid synthesized was analyzed by GC–MS.

### GC–MS measurement of amino acids

The pellets were concentrated and hydrolyzed yielding amounts of protein-derived animo acids, once almost the biomass has been synthesized from ^13^C labeled glucose. Pellets were disrupted into the well-sealed tubes containing 1.5 ml HCl (6 M) for 22 h in an oven at 110 °C. The only carbon source that is incorporated into amino acids should be glucose. Hydrolysate was then fractionated by a filter membrane with a pore size of 0.22 μm and it was evaporated under a constant steam of air at 80 °C. Samples need to be completely dry out. T-butyldimethylsilyl (TBDMS) of 100 μl was added for derivatization continuing 60 min at 85 °C after the dried residue redissolved in pyridine of 200 μl with sufficient mixing. The derivatized samples through previously centrifugation were then transferred into GC–MS vial and seal with caps.

GC–MS measurement was performed according the method described in the literature [26]. 1-μl derivatized sample was injected into a Shimadzu 2010 GC on a fused-silica capillary column (30.0 m × 250 nm × 0.25 μm). GC processes are as follows: initial temperature was set at 60 °C for 2 min, then increased to 180 °C at 5 °C per minute, and reached 260 °C for 5 min at 10 °C per minute. MS detecting conditions are as follows: vaporization and ion source temperature were respectively set at 250 °C and 230 °C, and the power voltage and detector voltage were respectively fitted at 70 eV and 350 V.

### Construction of a metabolic model for ^13^C metabolic flux analysis

Studying the metabolic network model is a prerequisite for performing ^13^C metabolic flux analysis. A metabolic network model of *M. circinelloides* was constructed on basis of validated models related to support. Concisely, the network covered all pivotal central pathways which primarily are the glycolysis, the TCA cycle and the PP pathway. In all flux ratio analysis, the model is complete when it describes the following aspects: (i) Stoichiometry. There must be incorporate all substrates, potential products, and detectable labeling intermediates in the stoichiometric model. Furthermore, all cofactors that devote to redox balance (such as NADH and NADPH) or energy equivalents (such as ATP or CoA) are traditionally ignored to ensure that their balance do not bias the flux estimates [20]. (ii) Carbon atom transitions. The metabolic and isotopic stability of cell culture are prerequisites for all current ^13^C metabolic flux analyses, which means that all intermediate concentrations and fluxes are constant throughout the ^13^C labeling experiment. (iii) Precursor requirements for biomass formation. In order to calculate absolute flux, the amount of metabolites extracted to implement biosynthesis requirements must be known. Determining the relative amounts of biomacromolecule such as carbohydrate, protein, DNA, RNA and lipids, the composition of the cell dry weight is obtained [36]. (iv) Finally, we highlight two assumptions implied in this model. On the one hand, enzymes does not distinguish between ^13^C or ^12^C atoms, and their activity is comparative for all isotopes. On the other hand, the isotopes in the metabolite pool are uniformly mixed during the process of enzymatic reaction.

### Metabolic modeling and flux analysis

Analyses were performed for electron impact (EI) mass spectrometry data of each chromatographic peak. The mass spectrum of each amino acid takes into account the sum of all scans of a chromatographic peak. The resulting EI mass spectral data is groups of ion clusters, each one describing the mass isotope distribution of a given amino acid fragment. An obviously detected fragments of the amino acid derivatized by TBDMS are shown in Additional file 3: Table S3 [37].

In this case, the mass isotope distribution vector (MDV) is distributed as follows [38]:

1 $${\text{MDV}}_{\upalpha} = \left[ {\begin{array}{*{20}c} {\left( {{\text{m}}_{0} } \right)} \\ {\left( {{\text{m}}_{1} } \right)} \\ \vdots \\ {\left( {{\text{m}}_{\text{n}} } \right)} \\ \end{array} } \right]\quad {\text{with}}\;\sum\limits_{{{\text{i}} = 0}}^{\text{n}} {{\text{m}}_{\text{i}} } = 1$$

where m_0_ represents the fractional abundance of the minimum mass and m_i_ > m_0_, i is the number of carbon atoms labeled.

The higher mass isotopic signals are derived from: (1) the natural abundance of non-C atoms (such as H, N, O, Si), (2) the natural abundance of ^13^C introduced by the derivative reagent, (3) the amino acid fragment of the natural or artificial ^13^C-labeled substrate. The ^13^C abundance in the amino acid carbon skeleton includes substrate- labeled and natural abundance [39].

A correction matrix MDV_α_ is used to correct the natural isotopic abundance of H, N, O, Si, S and C in the derivative so as to obtain dedicated mass isotope distribution of the carbon skeleton MDV_α_^*^. The natural isotopes abundances found in amino acids are summarized in Additional file 4: Table S4.

2 $${\text{MDV}}_{\upalpha}^{*} = {\text{C}}_{{{\text{corr}},{\text{CONHSiS}}}}^{ - 1} \cdot {\text{MDV}}_{\upalpha}$$

A correction formula is used to subtract contribution of ^13^C-unlabeled biomass yielding MDV_AA_ so as to obtain dedicated mass isotope distribution caused by artificially 13C-labeled substrates as follows [26]:

3 $${\text{MDV}}_{\text{AA}} = \frac{{{\text{MDV}}_{\upalpha}^{*} - {\text{f}}_{\text{unlabeled}} \cdot {\text{MDV}}_{{{\text{unlabeled}},{\text{n}}}} }}{{1 - {\text{f}}_{\text{unlabeled}} }}$$

For the [U-^13^C] glucose labeling experiment, The fractional label of the different derivatized fragments should be consistent with the labeling content of the input substrate which could be determined according to Eq 4.

4 $${\text{FL }} = \frac{{\sum\nolimits_{{{\text{i}} = 0}}^{{\text{n}}} {{\text{i}} \cdot {\text{m}}_{{\text{i}}} } }}{{{\text{n}} \cdot \sum\nolimits_{{{\text{i}} = 0}}^{{\text{n}}} {{\text{m}}_{{\text{i}}} } }}$$

### Flux determination and statistical analysis

In our study, metabolic fluxes and the confidence intervals were determined to simultaneously fit the external fluxes and the mass isotope abundances of intracellular amino acids to a detailed metabolic network model of *M. circinelloides* cells, which was performed using Metran [40]. The Metran software program was applied to ^13^C labeled metabolic flux analysis, tracer experimental design and statistical analysis. Each experiment was performed in triplicate. All data were represented as means ± standard deviations and were analyzed with ANOVA. Validation of the fit was accomplished by a χ^2^-statistical test for the goodness-of-fit between the experimental values of the mass isotopomers and those simulated from the estimated flux distribution [41].

## Supplementary information

**Additional file 1: Table S1.** Measured and estimated MDV of control and recombinant strains using proteinogenic amino acid.

**Additional file 2: Table S2.** Metabolic network model for ^13^C metabolic flux analysis.

**Additional file 3: Table S3.** The minimum mass spectrometric fragments of amino acids.

**Additional file 4: Table S4.** Abundance of natural stable isotopes for biologically relevant elements.

## Data Availability

All data generated or analyzed during this study are included in this published article (and its additional files).

## Abbreviations

GLA: gamma-linolenic acid
AMPD: adenosine monophosphate deaminase
AMP: adenosine monophosphate
ICDH: isocitrate dehydrogenase
HN: high nitrogen
LN: low nitrogen
DCW: dry cell weight
PP: pentose phosphate
TCA: tricarboxylic acid
GOX: glyoxylate
EMP: glycolytic pathway
IMP: inosine monophosphate
OAA: oxaloacetic acid
POM: pyruvate/oxaloacetate/malate
G6PD: glucose-6-phosphate dehydrogenase
6PGD: 6-phosphogluconate dehydrogenase
GOD: glucose oxidase–peroxidase
DNS: dinitrosalicylic acid
HPLC: high performance liquid chromatography
FAMEs: fatty acid methyl esters
TBDMS: T-butyldimethylsilyl
EI: electron impact
MDV: mass isotope distribution vector
