# Supplementary material for: 13C metabolic flux analysis on roles of malate transporter in lipid accumulation of Mucor circinelloides
Source: Microb Cell Fact. 2019 Sep 10;18:154. doi: 10.1186/s12934-019-1207-9 (PMC6737672; doi:10.1186/s12934-019-1207-9)
Supplement: Supplementary file 1 — Additional file 1: Table S1. Measured and estimated MDV of control and recombinant strains using proteinogenic amino acid. [file 12934_2019_1207_MOESM1_ESM.docx]

**Additional file 1**

Table S1 Measured and estimated MDV of control and recombinants using proteinogenic amino acid

| **Amino acid** | **Fragment** | **MDV** | **MU522-HN**  **measured** | **MU522-HN**  **estimated** | **MU241-HN**  **measured** | **MU241-HN**  **estimated** | **Mc-MT-1-HN**  **measured** | **Mc-MT-1-HN**  **estimated** | **Mc-MT-2-HN**  **measured** | **Mc-MT-2-HN**  **estimated** |
| --- | --- | --- | --- | --- | --- | --- | --- | --- | --- | --- |
| Ala | m_57 | M+0 | 0.7046 | 0.7150 | 0.7204 | 0.7208 | 0.7264 | 0.7257 | 0.7307 | 0.7277 |
|  |  | M+1 | 0.1015 | 0.0959 | 0.0972 | 0.0929 | 0.0848 | 0.0820 | 0.0826 | 0.0872 |
|  |  | M+2 | 0.0471 | 0.0488 | 0.0465 | 0.0472 | 0.0333 | 0.0393 | 0.0348 | 0.0322 |
|  |  | M+3 | 0.1468 | 0.1403 | 0.1459 | 0.1391 | 0.1556 | 0.1531 | 0.1519 | 0.1529 |
|  | m_85 | M+0 | 0.7639 | 0.7580 | 0.7536 | 0.7639 | 0.7585 | 0.7595 | 0.7624 | 0.7602 |
|  |  | M+1 | 0.0649 | 0.0659 | 0.0682 | 0.0649 | 0.0650 | 0.0679 | 0.0621 | 0.0665 |
|  |  | M+2 | 0.1712 | 0.1761 | 0.1683 | 0.1712 | 0.1765 | 0.1726 | 0.1755 | 0.1733 |
| Val | m_57 | M+0 | 0.5611 | 0.5368 | 0.5457 | 0.5396 | 0.5690 | 0.5674 | 0.5625 | 0.5725 |
|  |  | M+1 | 0.1255 | 0.1237 | 0.1275 | 0.1270 | 0.1281 | 0.1231 | 0.1336 | 0.1290 |
|  |  | M+2 | 0.1550 | 0.1671 | 0.1610 | 0.1622 | 0.1671 | 0.1648 | 0.1700 | 0.1666 |
|  |  | M+3 | 0.1322 | 0.1369 | 0.1289 | 0.1281 | 0.0959 | 0.1006 | 0.0945 | 0.0917 |
|  |  | M+4 | 0.0141 | 0.0157 | 0.0154 | 0.0189 | 0.0199 | 0.0194 | 0.0201 | 0.0201 |
|  |  | M+5 | 0.0122 | 0.0198 | 0.0216 | 0.0241 | 0.0202 | 0.0247 | 0.0193 | 0.0201 |
|  | m_85 | M+0 | 0.5787 | 0.5743 | 0.5613 | 0.5698 | 0.6003 | 0.6031 | 0.6067 | 0.6196 |
|  |  | M+1 | 0.1218 | 0.1316 | 0.1281 | 0.1170 | 0.1061 | 0.0989 | 0.1040 | 0.1040 |
|  |  | M+2 | 0.2419 | 0.2384 | 0.2543 | 0.2591 | 0.2415 | 0.2485 | 0.2379 | 0.2266 |
|  |  | M+3 | 0.0287 | 0.0289 | 0.0282 | 0.0260 | 0.0216 | 0.0218 | 0.0221 | 0.0216 |
|  |  | M+4 | 0.0289 | 0.0268 | 0.0280 | 0.0281 | 0.0305 | 0.0277 | 0.0293 | 0.0282 |
| Ile | m_85 | M+0 | 0.4479 | 0.4597 | 0.4507 | 0.4526 | 0.4998 | 0.4968 | 0.4876 | 0.4929 |
|  |  | M+1 | 0.2402 | 0.2417 | 0.2292 | 0.2217 | 0.2039 | 0.2079 | 0.2099 | 0.2179 |
|  |  | M+2 | 0.2193 | 0.2090 | 0.2262 | 0.2209 | 0.2272 | 0.2078 | 0.2180 | 0.2016 |
|  |  | M+3 | 0.0666 | 0.0640 | 0.0682 | 0.0736 | 0.0599 | 0.0608 | 0.0580 | 0.0600 |
|  |  | M+4 | 0.0207 | 0.0205 | 0.0205 | 0.0260 | 0.0248 | 0.0235 | 0.0239 | 0.0245 |
|  |  | M+5 | 0.0053 | 0.0051 | 0.0053 | 0.0052 | -0.0158 | 0.0032 | 0.0026 | 0.0031 |
|  | m_159 | M+0 | 0.4571 | 0.4547 | 0.4435 | 0.4526 | 0.4910 | 0.4968 | 0.4915 | 0.4830 |
|  |  | M+1 | 0.2264 | 0.2292 | 0.2238 | 0.2217 | 0.2059 | 0.2079 | 0.2017 | 0.2021 |
|  |  | M+2 | 0.2225 | 0.2243 | 0.2225 | 0.2209 | 0.2061 | 0.2078 | 0.2075 | 0.2130 |
|  |  | M+3 | 0.0682 | 0.0655 | 0.0759 | 0.0736 | 0.0657 | 0.0608 | 0.0669 | 0.0701 |
|  |  | M+4 | 0.0202 | 0.0209 | 0.0252 | 0.0260 | 0.0267 | 0.0235 | 0.0280 | 0.0272 |
|  |  | M+5 | 0.0056 | 0.0054 | 0.0090 | 0.0052 | 0.0046 | 0.0032 | 0.0044 | 0.0046 |
| Gly | m_57 | M+0 | 0.7067 | 0.7037 | 0.7125 | 0.7212 | 0.7342 | 0.7325 | 0.7409 | 0.7419 |
|  |  | M+1 | 0.1482 | 0.1483 | 0.1483 | 0.1445 | 0.1249 | 0.1223 | 0.1190 | 0.1210 |
|  |  | M+2 | 0.1451 | 0.1480 | 0.1392 | 0.1343 | 0.1409 | 0.1452 | 0.1401 | 0.1371 |
|  | m_85 | M+0 | 0.7927 | 0.7942 | 0.7931 | 0.7934 | 0.7963 | 0.7936 | 0.7893 | 0.7927 |
|  |  | M+1 | 0.2073 | 0.2058 | 0.2069 | 0.2066 | 0.2037 | 0.2064 | 0.2107 | 0.2073 |
| Ser | m_57 | M+0 | 0.7194 | 0.7115 | 0.7217 | 0.7260 | 0.7159 | 0.7287 | 0.7111 | 0.7098 |
|  |  | M+1 | 0.0935 | 0.0937 | 0.0904 | 0.0996 | 0.1068 | 0.1014 | 0.1076 | 0.1053 |
|  |  | M+2 | 0.0670 | 0.0689 | 0.0690 | 0.0683 | 0.0978 | 0.0910 | 0.1023 | 0.1021 |
|  |  | M+3 | 0.1201 | 0.1259 | 0.1190 | 0.1060 | 0.0794 | 0.0788 | 0.0790 | 0.0828 |
|  | m_85 | M+0 | 0.7601 | 0.7534 | 0.7718 | 0.7774 | 0.7654 | 0.7754 | 0.7697 | 0.7705 |
|  |  | M+1 | 0.0658 | 0.0648 | 0.1045 | 0.1014 | 0.1382 | 0.1358 | 0.1359 | 0.1304 |
|  |  | M+2 | 0.1741 | 0.1818 | 0.1236 | 0.1211 | 0.0964 | 0.0888 | 0.0944 | 0.0991 |
|  | m_159 | M+0 | 0.7582 | 0.7513 | 0.7666 | 0.7774 | 0.7778 | 0.7754 | 0.7582 | 0.7582 |
|  |  | M+1 | 0.1060 | 0.1108 | 0.1039 | 0.1014 | 0.1332 | 0.1358 | 0.1060 | 0.1060 |
|  |  | M+2 | 0.1358 | 0.1379 | 0.1295 | 0.1211 | 0.0890 | 0.0888 | 0.1358 | 0.1358 |
| Phe | m_57 | M+0 | 0.4035 | 0.4085 | 0.4137 | 0.4111 | 0.4038 | 0.4005 | 0.3940 | 0.3950 |
|  |  | M+1 | 0.1367 | 0.1414 | 0.1401 | 0.1369 | 0.1086 | 0.1164 | 0.1123 | 0.1151 |
|  |  | M+2 | 0.1291 | 0.1261 | 0.1240 | 0.1256 | 0.1309 | 0.1298 | 0.1300 | 0.1236 |
|  |  | M+3 | 0.1187 | 0.1144 | 0.1136 | 0.1153 | 0.1384 | 0.1368 | 0.1428 | 0.1466 |
|  |  | M+4 | 0.1112 | 0.1090 | 0.1067 | 0.1146 | 0.1122 | 0.1107 | 0.1162 | 0.1135 |
|  |  | M+5 | 0.0406 | 0.0390 | 0.0417 | 0.0483 | 0.0419 | 0.0447 | 0.0418 | 0.0424 |
|  |  | M+6 | 0.0262 | 0.0270 | 0.0259 | 0.0208 | 0.0357 | 0.0324 | 0.0355 | 0.0359 |
|  |  | M+7 | 0.0197 | 0.0202 | 0.0201 | 0.0184 | 0.0189 | 0.0210 | 0.0181 | 0.0186 |
|  |  | M+8 | 0.0092 | 0.0095 | 0.0089 | 0.0052 | 0.0055 | 0.0035 | 0.0054 | 0.0055 |
|  |  | M+9 | 0.0051 | 0.0049 | 0.0053 | 0.0038 | 0.0041 | 0.0041 | 0.0039 | 0.0038 |
|  | m_85 | M+0 | 0.4524 | 0.4483 | 0.4373 | 0.4751 | 0.4019 | 0.4183 | 0.4029 | 0.3931 |
|  |  | M+1 | 0.0934 | 0.0967 | 0.0924 | 0.1045 | 0.1189 | 0.1108 | 0.1172 | 0.1161 |
|  |  | M+2 | 0.2192 | 0.2209 | 0.2262 | 0.1890 | 0.2117 | 0.2104 | 0.2095 | 0.2131 |
|  |  | M+3 | 0.0680 | 0.0653 | 0.0713 | 0.0753 | 0.0691 | 0.0643 | 0.0706 | 0.0741 |
|  |  | M+4 | 0.1145 | 0.1153 | 0.1195 | 0.0908 | 0.1133 | 0.1173 | 0.1156 | 0.1199 |
|  |  | M+5 | 0.0182 | 0.0187 | 0.0174 | 0.0274 | 0.0292 | 0.0281 | 0.0285 | 0.0293 |
|  |  | M+6 | 0.0227 | 0.0233 | 0.0238 | 0.0308 | 0.0404 | 0.0418 | 0.0402 | 0.0387 |
|  |  | M+7 | 0.0073 | 0.0070 | 0.0076 | 0.0035 | 0.0089 | 0.0044 | 0.0088 | 0.0092 |
|  |  | M+8 | 0.0043 | 0.0045 | 0.0045 | 0.0036 | 0.0067 | 0.0047 | 0.0067 | 0.0065 |
|  | m_159 | M+0 | 0.4740 | 0.4654 | 0.4710 | 0.4751 | 0.4171 | 0.4183 | 0.4209 | 0.4312 |
|  |  | M+1 | 0.1003 | 0.0997 | 0.1021 | 0.1045 | 0.1054 | 0.1108 | 0.1013 | 0.0970 |
|  |  | M+2 | 0.1756 | 0.1841 | 0.1811 | 0.189 | 0.2205 | 0.2104 | 0.2216 | 0.2211 |
|  |  | M+3 | 0.0705 | 0.0670 | 0.0731 | 0.0753 | 0.0669 | 0.0643 | 0.0666 | 0.0650 |
|  |  | M+4 | 0.0947 | 0.0984 | 0.0906 | 0.0908 | 0.1115 | 0.1173 | 0.1117 | 0.1062 |
|  |  | M+5 | 0.0385 | 0.0377 | 0.0368 | 0.0274 | 0.0243 | 0.0281 | 0.0237 | 0.0228 |
|  |  | M+6 | 0.0353 | 0.0365 | 0.0346 | 0.0308 | 0.0458 | 0.0418 | 0.0458 | 0.0480 |
|  |  | M+7 | 0.0033 | 0.0033 | 0.0033 | 0.0035 | 0.0044 | 0.0044 | 0.0042 | 0.0044 |
|  |  | M+8 | 0.0078 | 0.0079 | 0.0075 | 0.0036 | 0.0040 | 0.0047 | 0.0042 | 0.0043 |
|  | M+302 | M+0 | 0.7601 | 0.7672 | 0.7732 | 0.7840 | 0.7538 | 0.7565 | 0.7535 | 0.7555 |
|  |  | M+1 | 0.0658 | 0.0667 | 0.0782 | 0.0704 | 0.0719 | 0.0737 | 0.0724 | 0.0715 |
|  |  | M+2 | 0.1741 | 0.1661 | 0.1486 | 0.1456 | 0.1742 | 0.1698 | 0.1741 | 0.1730 |
| Tyr | M+302 | M+0 | 0.7601 | 0.7657 | 0.7411 | 0.7544 | 0.7723 | 0.7684 | 0.7798 | 0.7875 |
|  |  | M+1 | 0.0658 | 0.0639 | 0.0808 | 0.0779 | 0.0653 | 0.0709 | 0.0646 | 0.0627 |
|  |  | M+2 | 0.1741 | 0.1704 | 0.1780 | 0.1677 | 0.1623 | 0.1607 | 0.1556 | 0.1498 |
| Asp | m_57 | M+0 | 0.5632 | 0.5742 | 0.5387 | 0.5444 | 0.5498 | 0.5441 | 0.5568 | 0.5572 |
|  |  | M+1 | 0.1306 | 0.1272 | 0.2167 | 0.2178 | 0.2163 | 0.2183 | 0.2189 | 0.2237 |
|  |  | M+2 | 0.2497 | 0.2418 | 0.1577 | 0.1547 | 0.1498 | 0.1549 | 0.1433 | 0.1370 |
|  |  | M+3 | 0.0288 | 0.0283 | 0.0750 | 0.0731 | 0.0734 | 0.0729 | 0.0704 | 0.0719 |
|  |  | M+4 | 0.0277 | 0.0285 | 0.0118 | 0.0100 | 0.0108 | 0.0099 | 0.0106 | 0.0102 |
|  | m_85 | M+0 | 0.7194 | 0.7182 | 0.6028 | 0.6000 | 0.5982 | 0.6010 | 0.5881 | 0.5893 |
|  |  | M+1 | 0.0935 | 0.0955 | 0.2412 | 0.2373 | 0.2402 | 0.2384 | 0.2477 | 0.2425 |
|  |  | M+2 | 0.0670 | 0.0700 | 0.1329 | 0.1283 | 0.1414 | 0.1401 | 0.1432 | 0.1477 |
|  |  | M+3 | 0.1201 | 0.1163 | 0.0232 | 0.0344 | 0.0202 | 0.0205 | 0.0210 | 0.0205 |
|  | M+302 | M+0 | 0.7217 | 0.7168 | 0.7195 | 0.7190 | 0.7085 | 0.7179 | 0.6956 | 0.6999 |
|  |  | M+1 | 0.1692 | 0.1744 | 0.1681 | 0.1663 | 0.1670 | 0.1517 | 0.1687 | 0.1705 |
|  |  | M+2 | 0.1091 | 0.1088 | 0.1124 | 0.1147 | 0.1304 | 0.1304 | 0.1357 | 0.1296 |
| Thr | m_57 | M+0 | 0.6198 | 0.6256 | 0.6137 | 0.6110 | 0.5590 | 0.5546 | 0.5499 | 0.5484 |
|  |  | M+1 | 0.1927 | 0.1887 | 0.1899 | 0.1894 | 0.2076 | 0.2138 | 0.2092 | 0.2150 |
|  |  | M+2 | 0.1144 | 0.1154 | 0.1112 | 0.1299 | 0.1540 | 0.1509 | 0.1588 | 0.1583 |
|  |  | M+3 | 0.0637 | 0.0608 | 0.0659 | 0.0614 | 0.0730 | 0.0710 | 0.0755 | 0.0719 |
|  |  | M+4 | 0.0094 | 0.0095 | 0.0092 | 0.0084 | 0.0063 | 0.0097 | 0.0066 | 0.0064 |
|  | M+302 | M+0 | 0.6669 | 0.6631 | 0.6603 | 0.6593 | 0.6014 | 0.6104 | 0.5949 | 0.6007 |
|  |  | M+1 | 0.1966 | 0.1974 | 0.2066 | 0.2042 | 0.2380 | 0.2331 | 0.2431 | 0.2355 |
|  |  | M+2 | 0.1122 | 0.1150 | 0.1085 | 0.1077 | 0.1377 | 0.1365 | 0.1394 | 0.1405 |
|  |  | M+3 | 0.0243 | 0.0245 | 0.0245 | 0.0288 | 0.0228 | 0.0200 | 0.0226 | 0.0233 |
| Glu | m_57 | M+0 | 0.4479 | 0.4414 | 0.4575 | 0.4523 | 0.4654 | 0.4694 | 0.4623 | 0.4727 |
|  |  | M+1 | 0.2402 | 0.2502 | 0.2252 | 0.2259 | 0.2183 | 0.2178 | 0.2241 | 0.2248 |
|  |  | M+2 | 0.2193 | 0.2155 | 0.2186 | 0.2155 | 0.2251 | 0.2201 | 0.2252 | 0.2144 |
|  |  | M+3 | 0.0666 | 0.0663 | 0.0677 | 0.0756 | 0.0633 | 0.0644 | 0.0604 | 0.0599 |
|  |  | M+4 | 0.0207 | 0.0214 | 0.0272 | 0.0241 | 0.0269 | 0.0248 | 0.0270 | 0.0272 |
|  |  | M+5 | 0.0053 | 0.0052 | 0.0038 | 0.0057 | 0.0010 | 0.0034 | 0.0010 | 0.0010 |
|  | m_85 | M+0 | 0.4869 | 0.4704 | 0.4908 | 0.4978 | 0.5156 | 0.5110 | 0.5171 | 0.5234 |
|  |  | M+1 | 0.2502 | 0.2568 | 0.2541 | 0.2543 | 0.2495 | 0.2471 | 0.2425 | 0.2358 |
|  |  | M+2 | 0.1926 | 0.1999 | 0.1852 | 0.1853 | 0.1794 | 0.1798 | 0.1840 | 0.1857 |
|  |  | M+3 | 0.0554 | 0.0574 | 0.0545 | 0.0506 | 0.0455 | 0.0504 | 0.0461 | 0.0448 |
|  |  | M+4 | 0.0149 | 0.0155 | 0.0154 | 0.0120 | 0.0101 | 0.0117 | 0.0103 | 0.0103 |
|  | m_159 | M+0 | 0.5195 | 0.5304 | 0.5032 | 0.4978 | 0.5205 | 0.5210 | 0.5130 | 0.5128 |
|  |  | M+1 | 0.2095 | 0.2038 | 0.2522 | 0.2543 | 0.2045 | 0.2014 | 0.2059 | 0.1957 |
|  |  | M+2 | 0.2165 | 0.2115 | 0.1820 | 0.1853 | 0.2197 | 0.2155 | 0.2249 | 0.2351 |
|  |  | M+3 | 0.0331 | 0.0323 | 0.0511 | 0.0506 | 0.0332 | 0.0404 | 0.0330 | 0.0330 |
|  |  | M+4 | 0.0214 | 0.0220 | 0.0115 | 0.0120 | 0.0221 | 0.0217 | 0.0232 | 0.0234 |
| Leu | m_57 | M+0 | 0.4569 | 0.4498 | 0.4545 | 0.4574 | 0.4746 | 0.4727 | 0.4687 | 0.4525 |
|  |  | M+1 | 0.2183 | 0.2229 | 0.2183 | 0.2085 | 0.1868 | 0.1963 | 0.1848 | 0.1903 |
|  |  | M+2 | 0.2190 | 0.2198 | 0.2207 | 0.2276 | 0.2231 | 0.2261 | 0.2287 | 0.2400 |
|  |  | M+3 | 0.0713 | 0.0715 | 0.0710 | 0.0733 | 0.0740 | 0.0714 | 0.0755 | 0.0733 |
|  |  | M+4 | 0.0291 | 0.0304 | 0.0301 | 0.0274 | 0.0330 | 0.0276 | 0.0335 | 0.0350 |
|  |  | M+5 | 0.0054 | 0.0056 | 0.0054 | 0.0058 | 0.0085 | 0.0060 | 0.0088 | 0.0089 |
| Met | m_57 | M+0 | 0.5382 | 0.5446 | 0.5368 | 0.5387 | 0.5303 | 0.5382 | 0.5198 | 0.5281 |
|  |  | M+1 | 0.2275 | 0.2221 | 0.2257 | 0.2221 | 0.2291 | 0.2249 | 0.2329 | 0.2321 |
|  |  | M+2 | 0.1486 | 0.1449 | 0.1522 | 0.1561 | 0.1596 | 0.1475 | 0.1628 | 0.1503 |
|  |  | M+3 | 0.0703 | 0.0723 | 0.0695 | 0.0724 | 0.0658 | 0.0787 | 0.0688 | 0.0785 |
|  |  | M+4 | 0.0123 | 0.0129 | 0.0127 | 0.0105 | 0.0158 | 0.0106 | 0.0123 | 0.0109 |
|  |  | M+5 | 0.0031 | 0.0032 | 0.0031 | 0.0001 | -0.0006 | 0.0001 | 0.0034 | 0.0001 |
|  | m_85 | M+0 | 0.5766 | 0.5766 | 0.5861 | 0.5885 | 0.5878 | 0.5925 | 0.5843 | 0.5955 |
|  |  | M+1 | 0.2499 | 0.2434 | 0.2423 | 0.2412 | 0.2409 | 0.2423 | 0.2485 | 0.2384 |
|  |  | M+2 | 0.1297 | 0.1343 | 0.1273 | 0.1295 | 0.1477 | 0.1411 | 0.1428 | 0.1412 |
|  |  | M+3 | 0.0349 | 0.0365 | 0.0355 | 0.0354 | 0.0201 | 0.0218 | 0.0210 | 0.0214 |
|  |  | M+4 | 0.0089 | 0.0092 | 0.0088 | 0.0054 | 0.0035 | 0.0022 | 0.0034 | 0.0035 |
|  | m_159 | M+0 | 0.5814 | 0.5827 | 0.5915 | 0.5935 | 0.5911 | 0.5945 | 0.6076 | 0.6102 |
|  |  | M+1 | 0.2512 | 0.2448 | 0.2467 | 0.2412 | 0.2447 | 0.2423 | 0.2438 | 0.2402 |
|  |  | M+2 | 0.1289 | 0.1342 | 0.1241 | 0.1295 | 0.1255 | 0.1411 | 0.1236 | 0.1259 |
|  |  | M+3 | 0.0316 | 0.0312 | 0.0311 | 0.0354 | 0.0248 | 0.0218 | 0.0241 | 0.0230 |
|  |  | M+4 | 0.0069 | 0.0071 | 0.0066 | 0.0004 | 0.0039 | 0.0002 | 0.0009 | 0.0007 |

| **Amino acid** | **Fragment** | **MDV** | **MU522-LN**  **measured** | **MU522-LN**  **estimated** | **MU241-LN**  **measured** | **MU241-LN**  **estimated** | **Mc-MT-1-LN**  **measured** | **Mc-MT-1-LN**  **estimated** | **Mc-MT-2-LN**  **measured** | **Mc-MT-2-LN**  **estimated** |
| --- | --- | --- | --- | --- | --- | --- | --- | --- | --- | --- |
| Ala | m_57 | M+0 | 0.7106 | 0.7123 | 0.7360 | 0.7300 | 0.7485 | 0.7475 | 0.7303 | 0.7314 |
|  |  | M+1 | 0.1072 | 0.1050 | 0.0838 | 0.0846 | 0.0841 | 0.0882 | 0.0760 | 0.0770 |
|  |  | M+2 | 0.0446 | 0.0434 | 0.0524 | 0.0520 | 0.0335 | 0.0336 | 0.0317 | 0.0331 |
|  |  | M+3 | 0.1376 | 0.1393 | 0.1278 | 0.1334 | 0.1339 | 0.1307 | 0.1620 | 0.1585 |
|  | m_85 | M+0 | 0.7562 | 0.7547 | 0.7443 | 0.7543 | 0.7289 | 0.7347 | 0.7807 | 0.7869 |
|  |  | M+1 | 0.0580 | 0.0581 | 0.0740 | 0.0729 | 0.0708 | 0.0718 | 0.0641 | 0.0619 |
|  |  | M+2 | 0.1858 | 0.1872 | 0.1817 | 0.1728 | 0.2003 | 0.1935 | 0.1552 | 0.1512 |
| Val | m_57 | M+0 | 0.5465 | 0.5530 | 0.5194 | 0.5172 | 0.5358 | 0.5241 | 0.5663 | 0.5628 |
|  |  | M+1 | 0.1225 | 0.1226 | 0.1192 | 0.1177 | 0.1419 | 0.1452 | 0.1415 | 0.1403 |
|  |  | M+2 | 0.1612 | 0.1546 | 0.1777 | 0.1848 | 0.1804 | 0.1868 | 0.1712 | 0.1742 |
|  |  | M+3 | 0.1318 | 0.1330 | 0.1452 | 0.1412 | 0.1012 | 0.1021 | 0.0790 | 0.0805 |
|  |  | M+4 | 0.0144 | 0.0143 | 0.0180 | 0.0185 | 0.0184 | 0.0193 | 0.0186 | 0.0192 |
|  |  | M+5 | 0.0236 | 0.0225 | 0.0205 | 0.0206 | 0.0223 | 0.0225 | 0.0234 | 0.0230 |
|  | m_85 | M+0 | 0.5427 | 0.5565 | 0.5667 | 0.5646 | 0.6298 | 0.6284 | 0.6460 | 0.6481 |
|  |  | M+1 | 0.1322 | 0.1259 | 0.1481 | 0.1449 | 0.0962 | 0.0962 | 0.0908 | 0.0930 |
|  |  | M+2 | 0.2729 | 0.2640 | 0.2276 | 0.2331 | 0.2217 | 0.2247 | 0.2159 | 0.2106 |
|  |  | M+3 | 0.0249 | 0.0256 | 0.0258 | 0.0262 | 0.0194 | 0.0189 | 0.0214 | 0.0212 |
|  |  | M+4 | 0.0273 | 0.0280 | 0.0318 | 0.0312 | 0.0329 | 0.0318 | 0.0259 | 0.0271 |
| Ile | m_85 | M+0 | 0.4864 | 0.4809 | 0.4738 | 0.4716 | 0.4564 | 0.4545 | 0.4699 | 0.4734 |
|  |  | M+1 | 0.1983 | 0.2081 | 0.2295 | 0.2334 | 0.2271 | 0.2229 | 0.2353 | 0.2329 |
|  |  | M+2 | 0.2113 | 0.2072 | 0.2087 | 0.2040 | 0.2188 | 0.2244 | 0.2042 | 0.2006 |
|  |  | M+3 | 0.0762 | 0.0758 | 0.0645 | 0.0673 | 0.0688 | 0.0702 | 0.0669 | 0.0661 |
|  |  | M+4 | 0.0221 | 0.0223 | 0.0177 | 0.0181 | 0.0254 | 0.0246 | 0.0233 | 0.0242 |
|  |  | M+5 | 0.0057 | 0.0057 | 0.0058 | 0.0056 | 0.0035 | 0.0034 | 0.0050 | 0.0028 |
|  | m_159 | M+0 | 0.4601 | 0.4679 | 0.4026 | 0.3883 | 0.5125 | 0.5117 | 0.4676 | 0.4505 |
|  |  | M+1 | 0.2396 | 0.2294 | 0.2673 | 0.2786 | 0.1876 | 0.1831 | 0.2084 | 0.2188 |
|  |  | M+2 | 0.1945 | 0.1948 | 0.2288 | 0.2327 | 0.1972 | 0.2014 | 0.2190 | 0.2272 |
|  |  | M+3 | 0.0692 | 0.0722 | 0.0757 | 0.0741 | 0.0699 | 0.0722 | 0.0772 | 0.0742 |
|  |  | M+4 | 0.0270 | 0.0264 | 0.0202 | 0.0208 | 0.0286 | 0.0275 | 0.0257 | 0.0250 |
|  |  | M+5 | 0.0096 | 0.0093 | 0.0054 | 0.0055 | 0.0042 | 0.0041 | 0.0067 | 0.0043 |
| Gly | m_57 | M+0 | 0.7369 | 0.7361 | 0.7044 | 0.6942 | 0.7339 | 0.7385 | 0.7334 | 0.7381 |
|  |  | M+1 | 0.1440 | 0.1437 | 0.1665 | 0.1734 | 0.1082 | 0.1058 | 0.1355 | 0.1367 |
|  |  | M+2 | 0.1191 | 0.1202 | 0.1291 | 0.1324 | 0.1579 | 0.1557 | 0.1311 | 0.1252 |
|  | m_85 | M+0 | 0.7986 | 0.7970 | 0.7650 | 0.7725 | 0.7683 | 0.7592 | 0.7577 | 0.7671 |
|  |  | M+1 | 0.2014 | 0.2030 | 0.2350 | 0.2275 | 0.2317 | 0.2408 | 0.2423 | 0.2329 |
| Ser | m_57 | M+0 | 0.7071 | 0.7091 | 0.7218 | 0.7267 | 0.7156 | 0.7087 | 0.7134 | 0.7140 |
|  |  | M+1 | 0.0895 | 0.0910 | 0.0805 | 0.0809 | 0.1057 | 0.1103 | 0.0984 | 0.0944 |
|  |  | M+2 | 0.0675 | 0.0700 | 0.0642 | 0.0649 | 0.1008 | 0.1024 | 0.1072 | 0.1079 |
|  |  | M+3 | 0.1359 | 0.1299 | 0.1335 | 0.1275 | 0.0779 | 0.0786 | 0.0810 | 0.0837 |
|  | m_85 | M+0 | 0.7652 | 0.7637 | 0.7411 | 0.7459 | 0.7703 | 0.7737 | 0.7703 | 0.7679 |
|  |  | M+1 | 0.0982 | 0.0996 | 0.0745 | 0.0755 | 0.1266 | 0.1276 | 0.1158 | 0.1199 |
|  |  | M+2 | 0.1366 | 0.1367 | 0.1844 | 0.1786 | 0.1031 | 0.0987 | 0.1139 | 0.1122 |
|  | m_159 | M+0 | 0.7598 | 0.7556 | 0.7405 | 0.7443 | 0.7610 | 0.7547 | 0.7665 | 0.7629 |
|  |  | M+1 | 0.1106 | 0.1104 | 0.1189 | 0.1179 | 0.1386 | 0.1454 | 0.1029 | 0.1081 |
|  |  | M+2 | 0.1296 | 0.1340 | 0.1406 | 0.1378 | 0.1004 | 0.0999 | 0.1306 | 0.1290 |
| Phe | m_57 | M+0 | 0.4096 | 0.4013 | 0.4158 | 0.4026 | 0.4238 | 0.4262 | 0.4175 | 0.4210 |
|  |  | M+1 | 0.1470 | 0.1511 | 0.1522 | 0.1585 | 0.0949 | 0.0980 | 0.1055 | 0.1012 |
|  |  | M+2 | 0.1313 | 0.1265 | 0.1112 | 0.1157 | 0.1181 | 0.1173 | 0.1171 | 0.1124 |
|  |  | M+3 | 0.1061 | 0.1078 | 0.1054 | 0.1077 | 0.1357 | 0.1303 | 0.1349 | 0.1357 |
|  |  | M+4 | 0.0985 | 0.1019 | 0.1012 | 0.0988 | 0.1246 | 0.1273 | 0.1232 | 0.1247 |
|  |  | M+5 | 0.0475 | 0.0498 | 0.0462 | 0.0478 | 0.0400 | 0.0390 | 0.0435 | 0.0454 |
|  |  | M+6 | 0.0252 | 0.0258 | 0.0300 | 0.0295 | 0.0365 | 0.0352 | 0.0328 | 0.0341 |
|  |  | M+7 | 0.0210 | 0.0215 | 0.0226 | 0.0237 | 0.0163 | 0.0164 | 0.0166 | 0.0164 |
|  |  | M+8 | 0.0092 | 0.0096 | 0.0101 | 0.0106 | 0.0054 | 0.0055 | 0.0051 | 0.0052 |
|  |  | M+9 | 0.0046 | 0.0047 | 0.0053 | 0.0051 | 0.0047 | 0.0048 | 0.0038 | 0.0039 |
|  | m_85 | M+0 | 0.4155 | 0.4282 | 0.4554 | 0.4656 | 0.3855 | 0.3741 | 0.4195 | 0.4202 |
|  |  | M+1 | 0.0980 | 0.1000 | 0.1062 | 0.1017 | 0.1251 | 0.1258 | 0.1066 | 0.1091 |
|  |  | M+2 | 0.2557 | 0.2438 | 0.2097 | 0.2008 | 0.2276 | 0.2380 | 0.1882 | 0.1876 |
|  |  | M+3 | 0.0739 | 0.0716 | 0.0670 | 0.0674 | 0.0596 | 0.0602 | 0.0660 | 0.0682 |
|  |  | M+4 | 0.1058 | 0.1042 | 0.1048 | 0.1058 | 0.1178 | 0.1178 | 0.1339 | 0.1291 |
|  |  | M+5 | 0.0151 | 0.0155 | 0.0200 | 0.0206 | 0.0309 | 0.0313 | 0.0264 | 0.0262 |
|  |  | M+6 | 0.0250 | 0.0258 | 0.0248 | 0.0257 | 0.0383 | 0.0377 | 0.0425 | 0.0430 |
|  |  | M+7 | 0.0069 | 0.0070 | 0.0079 | 0.0082 | 0.0080 | 0.0080 | 0.0103 | 0.0099 |
|  |  | M+8 | 0.0041 | 0.0039 | 0.0042 | 0.0042 | 0.0072 | 0.0071 | 0.0066 | 0.0067 |
|  | m_159 | M+0 | 0.4661 | 0.4592 | 0.5259 | 0.5206 | 0.4369 | 0.4409 | 0.4372 | 0.4300 |
|  |  | M+1 | 0.1005 | 0.0967 | 0.0897 | 0.0940 | 0.0902 | 0.0926 | 0.0882 | 0.0911 |
|  |  | M+2 | 0.1917 | 0.1951 | 0.1523 | 0.1516 | 0.2131 | 0.2025 | 0.2154 | 0.2267 |
|  |  | M+3 | 0.0838 | 0.0878 | 0.0604 | 0.0592 | 0.0684 | 0.0695 | 0.0689 | 0.0661 |
|  |  | M+4 | 0.0865 | 0.0886 | 0.0854 | 0.0872 | 0.1149 | 0.1148 | 0.1114 | 0.1070 |
|  |  | M+5 | 0.0314 | 0.0329 | 0.0356 | 0.0365 | 0.0238 | 0.0249 | 0.0249 | 0.0258 |
|  |  | M+6 | 0.0298 | 0.0295 | 0.0385 | 0.0386 | 0.0445 | 0.0465 | 0.0456 | 0.0448 |
|  |  | M+7 | 0.0031 | 0.0031 | 0.0033 | 0.0034 | 0.0045 | 0.0046 | 0.0040 | 0.0042 |
|  |  | M+8 | 0.0071 | 0.0071 | 0.0089 | 0.0089 | 0.0037 | 0.0037 | 0.0044 | 0.0043 |
|  | M+302 | M+0 | 0.7537 | 0.7506 | 0.7635 | 0.7550 | 0.7567 | 0.7624 | 0.7661 | 0.7616 |
|  |  | M+1 | 0.0832 | 0.0855 | 0.0610 | 0.0639 | 0.0656 | 0.0648 | 0.0641 | 0.0661 |
|  |  | M+2 | 0.1631 | 0.1639 | 0.1755 | 0.1811 | 0.1777 | 0.1728 | 0.1698 | 0.1723 |
| Tyr | M+302 | M+0 | 0.7471 | 0.7547 | 0.7836 | 0.7803 | 0.7435 | 0.7372 | 0.7430 | 0.7435 |
|  |  | M+1 | 0.0918 | 0.0914 | 0.0636 | 0.0663 | 0.0742 | 0.0742 | 0.0767 | 0.0742 |
|  |  | M+2 | 0.1611 | 0.1539 | 0.1528 | 0.1534 | 0.1823 | 0.1886 | 0.1803 | 0.1823 |
| Asp | m_57 | M+0 | 0.5595 | 0.5632 | 0.5439 | 0.5262 | 0.5553 | 0.5619 | 0.5495 | 0.5459 |
|  |  | M+1 | 0.1900 | 0.1306 | 0.1402 | 0.1465 | 0.2061 | 0.1973 | 0.2253 | 0.2280 |
|  |  | M+2 | 0.1574 | 0.2497 | 0.2598 | 0.2727 | 0.1479 | 0.1482 | 0.1350 | 0.1344 |
|  |  | M+3 | 0.0818 | 0.0288 | 0.0305 | 0.0298 | 0.0796 | 0.0820 | 0.0797 | 0.0814 |
|  |  | M+4 | 0.0113 | 0.0277 | 0.0256 | 0.0248 | 0.0111 | 0.0106 | 0.0105 | 0.0103 |
|  | m_85 | M+0 | 0.6070 | 0.6192 | 0.7206 | 0.7182 | 0.5768 | 0.5908 | 0.6183 | 0.6132 |
|  |  | M+1 | 0.2423 | 0.2359 | 0.0834 | 0.0955 | 0.2417 | 0.2305 | 0.2174 | 0.2165 |
|  |  | M+2 | 0.1269 | 0.1222 | 0.0692 | 0.0700 | 0.1603 | 0.1579 | 0.1447 | 0.1504 |
|  |  | M+3 | 0.0238 | 0.0227 | 0.1268 | 0.1163 | 0.0212 | 0.0208 | 0.0196 | 0.0199 |
|  | M+302 | M+0 | 0.7309 | 0.7206 | 0.7559 | 0.7646 | 0.7401 | 0.7399 | 0.6654 | 0.6627 |
|  |  | M+1 | 0.1518 | 0.1570 | 0.1510 | 0.1448 | 0.1469 | 0.1466 | 0.1888 | 0.1924 |
|  |  | M+2 | 0.1173 | 0.1224 | 0.0931 | 0.0906 | 0.1130 | 0.1135 | 0.1458 | 0.1449 |
| Thr | m_57 | M+0 | 0.6382 | 0.6316 | 0.6282 | 0.6190 | 0.5852 | 0.5941 | 0.5520 | 0.5597 |
|  |  | M+1 | 0.1788 | 0.1862 | 0.1698 | 0.1755 | 0.1925 | 0.1830 | 0.2206 | 0.2183 |
|  |  | M+2 | 0.1062 | 0.1079 | 0.1298 | 0.1308 | 0.1377 | 0.1394 | 0.1556 | 0.1501 |
|  |  | M+3 | 0.0680 | 0.0659 | 0.0615 | 0.0645 | 0.0778 | 0.0764 | 0.0655 | 0.0659 |
|  |  | M+4 | 0.0088 | 0.0084 | 0.0107 | 0.0102 | 0.0068 | 0.0071 | 0.0063 | 0.0060 |
|  | M+302 | M+0 | 0.6517 | 0.6669 | 0.7026 | 0.6631 | 0.6122 | 0.6175 | 0.6669 | 0.6213 |
|  |  | M+1 | 0.2037 | 0.1966 | 0.1735 | 0.1974 | 0.2077 | 0.2001 | 0.1966 | 0.2330 |
|  |  | M+2 | 0.1193 | 0.1122 | 0.1014 | 0.1150 | 0.1569 | 0.1591 | 0.1122 | 0.1257 |
|  |  | M+3 | 0.0253 | 0.0243 | 0.0225 | 0.0245 | 0.0232 | 0.0233 | 0.0243 | 0.0200 |
| Glu | m_57 | M+0 | 0.4784 | 0.4838 | 0.4226 | 0.4170 | 0.4919 | 0.4975 | 0.4388 | 0.4437 |
|  |  | M+1 | 0.1945 | 0.1931 | 0.2716 | 0.2748 | 0.2190 | 0.2187 | 0.2420 | 0.2436 |
|  |  | M+2 | 0.2313 | 0.2314 | 0.2126 | 0.2112 | 0.2093 | 0.2029 | 0.2243 | 0.2187 |
|  |  | M+3 | 0.0633 | 0.0604 | 0.0649 | 0.0679 | 0.0553 | 0.0556 | 0.0685 | 0.0683 |
|  |  | M+4 | 0.0289 | 0.0276 | 0.0231 | 0.0239 | 0.0234 | 0.0242 | 0.0255 | 0.0248 |
|  |  | M+5 | 0.0036 | 0.0037 | 0.0052 | 0.0052 | 0.0011 | 0.0011 | 0.0009 | 0.0009 |
|  | m_85 | M+0 | 0.4764 | 0.4814 | 0.4807 | 0.4706 | 0.5121 | 0.5194 | 0.4951 | 0.4755 |
|  |  | M+1 | 0.2469 | 0.2464 | 0.2583 | 0.2627 | 0.2594 | 0.2478 | 0.2577 | 0.2699 |
|  |  | M+2 | 0.2008 | 0.1943 | 0.1839 | 0.1890 | 0.1671 | 0.1707 | 0.1840 | 0.1929 |
|  |  | M+3 | 0.0597 | 0.0622 | 0.0608 | 0.0611 | 0.0517 | 0.0523 | 0.0519 | 0.0500 |
|  |  | M+4 | 0.0162 | 0.0157 | 0.0163 | 0.0166 | 0.0097 | 0.0098 | 0.0113 | 0.0117 |
|  | m_159 | M+0 | 0.5377 | 0.5438 | 0.4910 | 0.4930 | 0.4927 | 0.4901 | 0.5184 | 0.5121 |
|  |  | M+1 | 0.2237 | 0.2148 | 0.2259 | 0.2188 | 0.2016 | 0.2068 | 0.2135 | 0.2206 |
|  |  | M+2 | 0.1845 | 0.1865 | 0.2306 | 0.2348 | 0.2519 | 0.2499 | 0.2090 | 0.2084 |
|  |  | M+3 | 0.0295 | 0.0306 | 0.0343 | 0.0360 | 0.0344 | 0.0330 | 0.0367 | 0.0358 |
|  |  | M+4 | 0.0246 | 0.0243 | 0.0182 | 0.0174 | 0.0194 | 0.0202 | 0.0224 | 0.0231 |
| Leu | m_57 | M+0 | 0.4770 | 0.4903 | 0.4839 | 0.4712 | 0.5067 | 0.5237 | 0.4692 | 0.4585 |
|  |  | M+1 | 0.1993 | 0.1901 | 0.2119 | 0.2205 | 0.1920 | 0.1850 | 0.1939 | 0.1996 |
|  |  | M+2 | 0.2194 | 0.2147 | 0.2009 | 0.2064 | 0.1975 | 0.1890 | 0.2376 | 0.2392 |
|  |  | M+3 | 0.0695 | 0.0710 | 0.0650 | 0.0635 | 0.0664 | 0.0663 | 0.0627 | 0.0649 |
|  |  | M+4 | 0.0290 | 0.0279 | 0.0328 | 0.0331 | 0.0297 | 0.0283 | 0.0286 | 0.0301 |
|  |  | M+5 | 0.0058 | 0.0060 | 0.0055 | 0.0053 | 0.0077 | 0.0077 | 0.0080 | 0.0077 |
| Met | m_57 | M+0 | 0.5851 | 0.5855 | 0.5353 | 0.5275 | 0.5370 | 0.5002 | 0.5131 | 0.5123 |
|  |  | M+1 | 0.1959 | 0.1989 | 0.2373 | 0.2470 | 0.2133 | 0.1936 | 0.2362 | 0.2375 |
|  |  | M+2 | 0.1444 | 0.1390 | 0.1512 | 0.1501 | 0.1497 | 0.2028 | 0.1601 | 0.1610 |
|  |  | M+3 | 0.0608 | 0.0632 | 0.0601 | 0.0595 | 0.0742 | 0.0656 | 0.0798 | 0.0793 |
|  |  | M+4 | 0.0109 | 0.0105 | 0.0127 | 0.0124 | 0.0204 | 0.0299 | 0.0099 | 0.0098 |
|  |  | M+5 | 0.0029 | 0.0029 | 0.0034 | 0.0035 | 0.0054 | 0.0079 | 0.0009 | 0.0001 |
|  | m_85 | M+0 | 0.5729 | 0.5729 | 0.5402 | 0.5485 | 0.5887 | 0.6050 | 0.6025 | 0.5917 |
|  |  | M+1 | 0.2612 | 0.2614 | 0.2808 | 0.2674 | 0.2396 | 0.2278 | 0.2246 | 0.2332 |
|  |  | M+2 | 0.1228 | 0.1212 | 0.1343 | 0.1404 | 0.1488 | 0.1439 | 0.1471 | 0.1483 |
|  |  | M+3 | 0.0353 | 0.0367 | 0.0368 | 0.0357 | 0.0197 | 0.0201 | 0.0220 | 0.0228 |
|  |  | M+4 | 0.0078 | 0.0078 | 0.0079 | 0.0080 | 0.0032 | 0.0032 | 0.0038 | 0.0040 |
|  | m_159 | M+0 | 0.6367 | 0.6261 | 0.5991 | 0.5875 | 0.6135 | 0.6170 | 0.5618 | 0.5766 |
|  |  | M+1 | 0.2126 | 0.2235 | 0.2394 | 0.2452 | 0.2407 | 0.2389 | 0.2794 | 0.2576 |
|  |  | M+2 | 0.1169 | 0.1185 | 0.1225 | 0.1274 | 0.1211 | 0.1211 | 0.1311 | 0.1090 |
|  |  | M+3 | 0.0269 | 0.0261 | 0.0329 | 0.0335 | 0.0230 | 0.0220 | 0.0253 | 0.0212 |
|  |  | M+4 | 0.0045 | 0.0058 | 0.0061 | 0.0064 | 0.0017 | 0.0010 | 0.0034 | 0.0009 |
