# Supplementary material for: 13C metabolic flux analysis on roles of malate transporter in lipid accumulation of Mucor circinelloides
Source: Microb Cell Fact. 2019 Sep 10;18:154. doi: 10.1186/s12934-019-1207-9 (PMC6737672; doi:10.1186/s12934-019-1207-9)
Supplement: Supplementary file 2 — Additional file 2: Table S2. Metabolic network model for 13C metabolic flux analysis. [file 12934_2019_1207_MOESM2_ESM.docx]

**Additional file 2**

Table S2 Metabolic network model for ^13^C metabolic flux analysis.

| Flux_number | Reaction | Carbon atom transitions |
| --- | --- | --- |
| r1 | Gluc + ATP --> G6P + ADP | abcdef --> abcdef |
| r2 | G6P --> F6P | abcdef --> abcdef |
| r3 | F6P + ATP --> FBP | abcdef --> abcdef |
| r4 | FBP --> GAP + DHAP | abcdef --> cba + def |
| r5 | DHAP --> GAP | abc --> abc |
| r6 | GAP + Pi + ADP + NAD --> 3PG + ATP + NADH | abc --> abc |
| r7 | 3PG --> PEP | abc --> abc |
| r8 | PEP --> Pyr + ATP | abc --> abc |
| r9 | G6P + ATP --> ADP + ADPG | abcdef --> abcdef |
| r10 | Cit + ATP --> AcCoA + OAA + ADP | abcdef --> ef + abcd |
| r11 | G6P + NADP --> 6PG + NADPH | abcdef --> abcdef |
| r12 | 6PG + NADP --> Ru5P + NADPH + CO_2_ | abcdef --> bcdef + a |
| r13 | Ru5P --> X5P | abcde --> abcde |
| r14 | Ru5P --> R5P | abcde --> abcde |
| r15 | X5P + R5P --> GAP + S7P | abcde + fghij --> hij + fgabcde |
| r16 | S7P + GAP --> E4P + F6P | abcedfg + hij --> defg + abchij |
| r17 | E4P + X5P --> F6P + GAP | abcd +efghi --> efabcd + ghi |
| r18 | Pyr + NAD -> AcCoA + NADH + CO_2_ | abc --> bc + a |
| r19 | AcCoA + OAA --> Cit | ef + abcd --> dcbfea |
| r20 | Cit --> ICit | abcdef --> abcdef |
| r21 | ICit + NAD(P) --> AKG + NAD(P)H + CO_2_ | abcdef --> abcde + f |
| r22 | AKG + NAD --> SucCoA + CO_2_ + NADH | abcde --> bcde + a |
| r23 | SucCoA + ADP + Pi --> Suc + ATP | abcd --> ^1^/_2_ abcd + ^1^/_2_ dcba |
| r24 | Suc + FAD --> Fum + FADH_2_ | ^1^/_2_ abcd + ^1^/_2_ dcba --> ^1^/_2_ abcd + ^1^/_2_ dcba |
| r25 | Fum --> Mal | ^1^/_2_ abcd + ^1^/_2_ dcba --> abcd |
| r26 | Mal + NAD --> OAA + NADH | abcd --> abcd |
| r27 | ICit --> Glyox + Suc | abcdef --> ab + ^1^/_2_ edcf + ^1^/_2_ fcde |
| r28 | Glyox + AcCoA--> Mal | ab + cd --> abdc |
| r29 | Mal + NADP --> Pyr + NADPH + CO_2_ | abcd --> abc + d |
| r30 | PEP + CO_2_--> OAA + Pi | abc + d --> abcd |
| r31 | OAA + ATP --> PEP + ADP + CO_2_ | abcd --> abc + d |
| r32 | Pyr + CO_2_ + ATP --> OAA + ADP + Pi | abc + d --> abcd |
| r33 | OAA + GTP --> PEP + GDP + CO_2_ | abcd --> abc + d |
| r34 | DHAP + NADH --> Glyc3P+ NAD | abc --> abc |
| r35 | 7 AcCoA + 12 NADPH + 6 ATP --> C14:0 + 12 NADH + 6 ADP+6 Pi | ab + cd + ef + gh + ij + kl + mn --> abcdefghijklmn |
| r36 | 8 AcCoA + 14 NADPH + 7 ATP --> C16:0 + 14 NADH + 7 ADP + 7 Pi | ab + cd + ef + gh + ij + kl + mn + op --> abcdefghijklmnop |
| r37 | C16:0 + NADH + O_2_ --> C16:1 + NAD | abcdefghijklmnop --> abcdefghijklmnop |
| r38 | 9 AcCoA + 16 NADPH + 8 ATP --> C18:0 + 16 NADH + 8 ADP + 8 Pi | ab + cd + ef + gh + ij + kl + mn + op + qr --> abcdefghijklmnoprs |
| r39 | C18:0 + NADH + O_2_ --> C18:1 + NAD | abcdefghijklmnoprs --> abcdefghijklmnoprs |
| r40 | C18:1 + NADH + O_2_ --> C18:2 + NAD | abcdefghijklmnoprs --> abcdefghijklmnoprs |
| r41 | C18:2 + NADH + O_2_ --> C18:3 + NAD | abcdefghijklmnoprs --> abcdefghijklmnoprs |
| r42 | AKG + NADPH + NH_3_ --> Glu + NADP | abcde --> abcde |
| r43 | Glu + NH_3_ + ATP --> Gln + ADP + Pi | abcde --> abcde |
| r44 | Glu + ATP + 2 NADPH --> Pro + ADP + Pi + 2 NADP | abcde --> abcde |
| r45 | Glu + CO_2_ + Gln + Asp + NADPH + AcCoA + 5 ATP --> Arg + AKG + NADP + Fum + Ac + 5 ADP + 5 Pi | abcde + f + ghijk + lmno + pq --> abcdef + ghijk + lmno + pq |
| r46 | OAA + Glu --> Asp + AKG | abcd +efghi --> abcd + efghi |
| r47 | Asp + 2 ATP + NH_3_ --> Asn + 2 ADP + 2 Pi | abcd --> abcd |
| r48 | Pyr + Glu --> Ala + AKG | abc + defgh --> abc + defgh |
| r49 | 3PG + Glu + NAD --> Ser + NADH + AKG + Pi | abc + defgh --> abc + defgh |
| r50 | Ser + THF --> Gly + MEETHF | abc --> ab + c |
| r51 | Gly + THF + NAD --> MEETHF + NH_3_ + NADH + CO_2_ | ab --> b + a |
| r52 | Thr + NAD --> Gly + AcCoA + NADH | abcd --> ab + cd |
| r53 | Ser + AcCoA + SO_4_ + 4 NADPH + 3 ATP --> Cys + Ac + 3 ADP + 3 Pi + 4 NADP | abc + de --> abc + de |
| r54 | Asp + Pyr + Glu + SucCoA + 2 NADPH + ATP --> LL-DAP + AKG + Suc + 2 NADP + ADP + Pi | abcd + efg +hijkl + mnop --> ^1^/_2_ abcdgfe + ^1^/_2_ efgdcba + hijkl + ^1^/_2_ mnop + ^1^/_2_ ponm |
| r55 | LL-DAP --> Lys + CO_2_ | ^1^/_2_ abcdgfe + ^1^/_2_ gfedcba --> abcdef + g |
| r56 | Asp + 2 ATP + 2 NADPH --> Thr + 2 ADP + 2 Pi + 2 NADP | abcd --> abcd |
| r57 | Asp + METHF + Cys + SucCoA + ATP + 2 NADPH --> Met + Pyr + ADP + Pi + 2 NADP + Suc + NH_3_ + THF | abcd + e + fgh +ijkl --> abcde + fgh + ^1^/_2_ ijkl + ^1^/_2_ lkji |
| r58 | 2 Pyr + Glu + NADPH --> Val + AKG + NADP + CO_2_ | abc + def + ghijk --> abcef + ghijk + d |
| r59 | AcCoA + 2 Pyr + Glu + NAD + NADPH --> Leu + 2 CO_2_+ AKG + NADH + NADP | ab + cde + fgh + ijklm --> abdghe + c + f + ijklm |
| r60 | Thr + Pyr + Glu + NADPH --> Ile + CO_2_ + AKG + NADP + NH_3_ | abcd + efg + hijkl --> abfcdg + e + hijkl |
| r61 | 2 PEP + E4P + Glu + NAD + ATP + NADPH --> Tyr + AKG + NADH + ADP + 4 Pi + NADP + CO_2_ | abc + def + ghij + klmno --> abcefghij + klmno + d |
| r62 | 2 PEP + E4P + Glu + ATP + NADPH --> Phe + AKG + ADP + 4 Pi + NADP + CO_2_ | abc + def + ghij + klmno --> abcefghij + klmno + d |
| r63 | Ser + R5P + PEP + E4P + PEP + Gln + 3 ATP + NADPH --> Trp + CO_2_ + GAP + Pyr + Glu + 3 ADP + 6 Pi + NADP | abc + defgh + ijk + lmno + pqr + stuvw --> abcedklmnoj + i + fgh + pqr + stuvw |
| r64 | R5P + FTHF + Gln + Asp + 2 NAD + 5 ATP --> His + AKG + THF + Fum + 2 NADH + 5 ADP + 6 Pi | abcde + f + ghijk + lmno --> wdcbaf + ghijk + lmno |
| r65 | FADH_2_ + 2 ADP + 2 Pi + 0.5 O_2_ --> FAD + 1.5 ATP |  |
| r66 | NADH + 3 ADP + 3 Pi + 0.5 O_2_ --> NAD + 2.5 ATP |  |
| r67 | NADH + NADP --> NADPH + NAD |  |
| r68 | 2 ATP + Gln + CO2 --> 2 ADP + Glu + Car P | abcde --> abcde |
| r69 | R5P + ATP --> PRPP + AD | abcde --> abcde |
| r70 | 4 ATP + PRPP + Asp + 2 Gln + Gly + 2 FTHF + CO_2_ --> 4ADP +Fum + 2 Glu + IMP + 2 THF | abcde + fghi +2(jklmn) + op + q+ r + s --> fghi + 2(jklmn) + abcdeqopsr |
| r71 | 3 ATP + Gln + IMP + NAD --> 3 ADP + Glu + r GTP + NADH | abcde + fghijklmno --> abcde + fghijklmno |
| r72 | 3 ATP + Asp + IMP --> Fum + r ATP | abcd + efghijklmn --> abcd + efghijklmn |
| r73 | Car P + Asp + PRPP + NAD + 2 ATP --> r UTP + CO_2_ + NADH + 2 ADP | a + bcde + fghij --> fghijcdea + b |
| r74 | Gln + r UTP + ATP --> Glu + rCTP + ADP | abcde + figklmnop --> abcde + figklmnop |
| r75 | d CTP + METHF --> d TTP + THF + NH_3_ | abcdefghi + j --> abcdefgjhi |
| r76 | r CTP + NADPH --> d CTP + NADP | abcdefghi --> abcdefghi |
| r77 | r GTP + NADPH --> d GTP + NADP | abcdefghij --> abcdefghij |
| r78 | r ATP + NADPH --> d ATP + NADP | abcdefghij --> abcdefghij |
| r79 | 0.223 Asp + 0.223 Asn + 0.278 Glu + 0.278 Gln + 0.090 Ser + 0.046 His + 0.204 Gly + 0.107 Thr + 0.096 Arg + 0.300 Ala + 0.040 Tyr +0.004 Cys + 0.152 Val + 0.045 Met + 0.078 Phe + 0.111 Ile + 0.154 Leu +0.153 Lys + 0.078 Pro + 0.006 C14:0 + 0.036 C16:0 + 0.005 C16:1 + 0.005 C18:0 + 0.022 C18:1 + 0.030 C18:2 + 0.041 C18:3 + 0.147 Glyc3P + 0.654 ADPG + 19.776 ATP + 0.035 dATP + 0.035 dTTP + 0.025 dGTP + 0.025 dCTP + 0.074 rATP + 0. 074 rUTP + 0.054 rGTP + 0.054 rCTP --> Biomass + 19.776 ADP (MU522-HN) |  |
| r80 | 0.112 Asp + 0.112 Asn + 0.123 Glu + 0.123 Gln + 0.044 Ser + 0.022 His + 0.093 Gly + 0.050 Thr + 0.040 Arg + 0.102 Ala + 0.018 Tyr +0.002 Cys + 0.067 Val + 0.017 Met + 0.037 Phe + 0.052 Ile + 0.072 Leu +0.066 Lys + 0.037 Pro + 0.016 C14:0 + 0.040 C16:0 + 0.007 C16:1 + 0.013 C18:0 + 0.025 C18:1 + 0.019 C18:2 + 0.038 C18:3 + 0.158 Glyc3P + 1.523 ADPG + 14.503 ATP + 0.015 dATP + 0.015 dTTP + 0.011 dGTP + 0.011 dCTP + 0.045 rATP + 0.045 rUTP + 0.033 rGTP + 0.033 rCTP --> Biomass + 14.503 ADP (MU522-LN) |  |
| r81 | 0.207 Asp + 0.207 Asn + 0.249 Glu + 0.249 Gln + 0.086 Ser + 0.041 His + 0.194 Gly + 0.099 Thr + 0.091Arg + 0.252 Ala + 0.036 Tyr +0.003 Cys + 0.143 Val + 0.040 Met + 0.073 Phe + 0.105 Ile + 0.147 Leu +0.149 Lys + 0.087 Pro + 0.005 C14:0 + 0.045 C16:0 + 0.004 C16:1 + 0.007 C18:0 + 0.020 C18:1 + 0.026 C18:2 + 0.058 C18:3 + 0.166 Glyc3P + 0.433 ADPG + 19.077 ATP + 0.022 dATP + 0.022 dTTP + 0.016 dGTP + 0.016 dCTP + 0.081 rATP + 0.081 rUTP + 0.059 rGTP + 0.059 rCTP --> Biomass + 19.077 ADP (MU241-HN) |  |
| r82 | 0.093 Asp + 0.093 Asn + 0.108 Glu + 0.108 Gln + 0.038 Ser + 0.018 His + 0.083 Gly + 0.039 Thr + 0.035 Arg + 0.094 Ala + 0.020 Tyr +0.001 Cys + 0.071 Val + 0.016 Met + 0.031 Phe + 0.046 Ile + 0.064 Leu +0.057 Lys + 0.054 Pro + 0.006 C14:0 + 0.048 C16:0 + 0.004 C16:1 + 0.006 C18:0 + 0.024 C18:1 + 0.033 C18:2 + 0.061 C18:3 + 0.183 Glyc3P + 1.703 ADPG + 14.286 ATP + 0.018 dATP + 0.018 dTTP + 0.013 dGTP + 0.013 dCTP + 0.038 rATP + 0.038 rUTP + 0.028 rGTP + 0.028 rCTP --> Biomass + 14.286 ADP (MU241-LN) |  |
| r83 | 0.253 Asp + 0.253 Asn + 0.215 Glu + 0.215 Gln + 0.074 Ser + 0.040 His + 0.158 Gly + 0.086 Thr + 0.101 Arg + 0.185 Ala + 0.031 Tyr +0.003 Cys + 0.120 Val + 0.034 Met + 0.063 Phe + 0.090 Ile + 0.127 Leu +0.152 Lys + 0.077 Pro + 0.006 C14:0 + 0.038 C16:0 + 0.007 C16:1 + 0.006 C18:0 + 0.032 C18:1 + 0.035 C18:2 + 0.050 C18:3 + 0.173 Glyc3P + 0.584 ADPG + 18.417 ATP + 0.062 dATP + 0.062 dTTP + 0.046 dGTP + 0.046 dCTP + 0.075 rATP + 0.075 rUTP + 0.054 rGTP + 0.054 rCTP --> Biomass + 18.417 ADP (Mc-MT-1-HN) |  |
| r84 | 0.155 Asp + 0.155 Asn + 0.148 Glu + 0.148 Gln + 0.050 Ser + 0.029 His + 0.108 Gly + 0.059 Thr + 0.056 Arg + 0.153 Ala + 0.021 Tyr +0.003 Cys + 0.081 Val + 0.022 Met + 0.041 Phe + 0.061 Ile + 0.085 Leu +0.103 Lys + 0.050 Pro + 0.016 C14:0 + 0.050 C16:0 + 0.009 C16:1 + 0.017 C18:0 + 0.032 C18:1 + 0.024 C18:2 + 0.047 C18:3 + 0.197 Glyc3P + 1.629 ADPG + 16.017 ATP + 0.042 dATP + 0.042 dTTP + 0.031 dGTP + 0.031 dCTP + 0.051 rATP + 0.051 rUTP + 0.037 rGTP + 0.037 rCTP --> Biomass + 16.017 ADP (Mc-MT-1-LN) |  |
| r85 | 0.150 Asp + 0.150 Asn + 0.181 Glu + 0.181 Gln + 0.056 Ser + 0.027 His + 0.123 Gly + 0.065 Thr + 0.065 Arg + 0.157 Ala + 0.025 Tyr +0.003 Cys + 0.091 Val + 0.026 Met + 0.046 Phe + 0.094 Ile + 0.069 Leu +0.099 Lys + 0.055 Pro + 0.003 C14:0 + 0.045 C16:0 + 0.002 C16:1 + 0.010 C18:0 + 0.037 C18:1 + 0.032 C18:2 + 0.046 C18:3 + 0.174 Glyc3P + 0.740 ADPG + 16.212 ATP + 0.025 dATP + 0.025 dTTP + 0.018 dGTP + 0.018 dCTP + 0.055 rATP + 0.055 rUTP + 0.040 rGTP + 0.040 rCTP --> Biomass + 16.212ADP (Mc-MT-2-HN) |  |
| r86 | 0.150 Asp + 0.150 Asn + 0.161 Glu + 0.161 Gln + 0.053 Ser + 0.026 His + 0.116 Gly + 0.063 Thr + 0.061 Arg + 0.140 Ala + 0.025 Tyr +0.004 Cys + 0.087 Val + 0.025 Met + 0.043 Phe + 0.065 Ile + 0.088 Leu +0.100 Lys + 0.047 Pro + 0.005 C14:0 + 0.054 C16:0 + 0.003 C16:1 + 0.014 C18:0 + 0.043 C18:1 + 0.031 C18:2 + 0.051 C18:3 + 0.201 Glyc3P + 1.640 ADPG + 16.097 ATP + 0.029 dATP + 0.029 dTTP + 0.021 dGTP + 0.021 dCTP + 0.053 rATP + 0.053 rUTP + 0.038 rGTP + 0.038 rCTP --> Biomass + 16.097 ADP (Mc-MT-2-LN) |  |
