# Supplementary material for: 13C metabolic flux analysis on roles of malate transporter in lipid accumulation of Mucor circinelloides
Source: Microb Cell Fact. 2019 Sep 10;18:154. doi: 10.1186/s12934-019-1207-9 (PMC6737672; doi:10.1186/s12934-019-1207-9)
Supplement: Supplementary file 3 — Additional file 3: Table S3. The minimum mass spectrometric fragments of amino acids. [file 12934_2019_1207_MOESM3_ESM.docx]

**Additional file 3**

Table S3: The minimum mass spectrometric fragments of amino acids

| Amino acid | (M-15)^+^ | (M-57)^+^ | (M-85)^+^ | (M-159)^+^ | (f302)^+^ |
| --- | --- | --- | --- | --- | --- |
| Alanine |  | 260(3) | 232(2) |  |  |
| Glycine | 288(2) | 246(2) | 218(1) | 144(1) |  |
| Valine |  | 288(5) | 260(4) |  | 302(2) |
| Leucine | 344(6) |  | 274(5) | 200(5) |  |
| Isoleucine | 344(6) |  | 274(5) | 200(5) |  |
| Proline | 328(5) | 286(5) | 258(4) | 184(4) |  |
| Methionine |  | 320(5) | 292(4) | 218(4) |  |
| Serine | 432(3) | 390(3) | 362(2) | 288(2) | 302(2) |
| Threonine | 446(4) | 404(4) | 376(3) |  |  |
| Phenylalanine |  | 336(9) | 308(8) | 234(8) | 302(2) |
| Aspartate | 460(4) | 418(4) | 390(3) | 316(3) | 302(2) |
| Glutamate | 474(5) | 432(5) | 404(4) | 330(4) | 302(2) |
| Lysine |  | 431(6) |  | 329(5) |  |
| Histidine | 482(6) | 440(6) | 412(5) | 338(5) | 302(2) |
| Tyrosine | 508(9) | 466(9) | 438(8) | 364(8) | 302(2) |
