# Supplementary material for: 13C metabolic flux analysis on roles of malate transporter in lipid accumulation of Mucor circinelloides
Source: Microb Cell Fact. 2019 Sep 10;18:154. doi: 10.1186/s12934-019-1207-9 (PMC6737672; doi:10.1186/s12934-019-1207-9)
Supplement: Supplementary file 4 — Additional file 4: Table S4. Abundance of natural stable isotopes for biologically relevant elements. [file 12934_2019_1207_MOESM4_ESM.docx]

**Additional file 4**

Table S4: Abundance of natural stable isotopes for biologically relevant elements

| Elements | | Mostly occurring mass (m_0_) | Abundance of m_0_ | Abundance of m_1_ | Abundance of m_2_ |
| --- | --- | --- | --- | --- | --- |
| H | 1 | | 0.9998885 | 0.000115 |  |
| C | 12 | | 0.9893 | 0.0107 |  |
| N | 14 | | 0.99632 | 0.00368 |  |
| O | 16 | | 0.99757 | 0.00038 | 0.00205 |
| Si | 28 | | 0.922297 | 0.046832 | 0.030872 |
